# CK2-mediated HDAC5 shuttling regulates DNA end resection through Ku70 deacetylation

**DOI:** 10.7150/thno.122935

**Published:** 2026-01-08

**Authors:** Xueyi Liang, Jingyuan Zhao, Shoukang Li, Ruozheng Wei, Haixin Yu, Qiyue Zhang, Qixun Fu, Gengdu Qin, Yuhan Zhao, Jiaying Liu, Zhiqiang Liu, Tao Peng, Junpeng Meng, Shanmiao Gou, Tao Yin, Heshui Wu, Bo Wang, Yingke Zhou

**Affiliations:** 1 Department of Pancreatic Surgery, Union Hospital, Tongji Medical College, Huazhong University of Science and Technology, Wuhan 430022, Hubei, China.; 2 Sino-German Laboratory of Personalized Medicine for Pancreatic Cancer, Union Hospital, Tongji Medical College, Huazhong University of Science and Technology, Wuhan 430022, Hubei, China.; 3 Department of Thoracic Surgery, Union Hospital, Tongji Medical College, Huazhong University of Science and Technology, Wuhan 430022, Hubei, China.; 4 Cancer Center, Union Hospital, Tongji Medical College, Huazhong University of Science and Technology, Wuhan 430022, Hubei, China.; 5 Department of Pathology, Union Hospital, Tongji Medical College, Huazhong University of Science and Technology, Wuhan 430022, Hubei, China.; 6 Department of General Surgery, The Second Hospital of Shanxi Medical University, Taiyuan, China.

**Keywords:** pancreatic ductal adenocarcinoma, HDAC5, CK2, Ku70, PARP inhibitor

## Abstract

**Rationale:** Loss of histone deacetylase 5 (HDAC5) is frequently observed in multiple malignancies, including pancreatic ductal adenocarcinoma (PDAC), and is associated with poor patient survival. Although HDAC5 has been implicated in DNA damage repair, the molecular mechanisms by which it regulates DNA double-strand break (DSB) repair pathway choice remain unclear.

**Methods:** Using PDAC cell lines, genetically engineered mouse models, patient-derived organoids, and biochemical assays, we investigated the role of HDAC5 in DNA end resection and homologous recombination (HR). Protein interactions, post-translational modifications, DNA repair pathway activity, and cellular responses to DNA damage and PARP inhibition were systematically analyzed.

**Results:** We identify HDAC5 as a critical regulator of DNA end resection and HR through deacetylation of Ku70. DNA damage induces casein kinase 2 (CK2)-mediated phosphorylation of HDAC5, promoting its nuclear translocation. Nuclear HDAC5 directly deacetylates Ku70 at lysine 287, facilitating Ku70 dissociation from DSB sites, thereby enabling DNA end resection and HR repair. In contrast, HDAC5 loss or CK2 inhibition results in Ku70 K287 hyperacetylation, prolonged retention of the Ku heterodimer at DSBs, impaired DNA end resection, and suppression of HR. Consequently, HDAC5-deficient PDAC cells exhibit increased sensitivity to PARP inhibitors, while pharmacological CK2 inhibition sensitizes HDAC5-proficient tumors to PARP inhibition.

**Conclusions:** These findings uncover a previously unrecognized CK2-HDAC5-Ku70 signaling axis that governs DNA repair pathway choice by regulating DNA end resection. Targeting this axis provides a mechanistic rationale for enhancing PARP inhibitor sensitivity in PDAC, including tumors without classical homologous recombination deficiency.

## Introduction

Ku70, a core component of the non-homologous end joining (NHEJ)pathway, plays a central role in determining the choice between DNA double-strand break (DSB) repair pathways. Upon DSB induction, the Ku70/Ku80 heterodimer is rapidly recruited to DNA ends, where it binds and protects them from nucleolytic processing, thereby favoring NHEJ-mediated repair 1-3. In contrast, the dissociation of Ku70 from DSB sites, or its regulation by post-translational modifications such as ubiquitination, facilitates DNA end resection, leading to the generation of single-stranded DNA (ssDNA) regions and the subsequent recruitment of RPA and RAD51, which promotes homologous recombination (HR) repair 4. In addition to ubiquitination, lysine acetylation of Ku70 and Ku80 has been identified as an important post-translational modification regulating the function of the Ku heterodimer 5-7. Ku70 acetylation has been associated with an enhanced DNA damage response and reduced apoptosis 8, 9. However, whether and how Ku70 acetylation influences its role in DNA end resection remains unclear.

Casein kinase 2 (CK2) is a ubiquitously expressed serine/threonine kinase that regulates a wide range of cellular processes, including DNA damage repair 10, 11. CK2 has been shown to be essential for NHEJ by activating DNA-dependent protein kinase (DNA-PK) 12. Moreover, CK2 inhibition has been reported to sensitize CBX3-deficient prostate cancer cells to PARP inhibitors 13. Together, these observations suggest that CK2 may play a previously underappreciated yet critical role in the regulation of NHEJ.

As a member of the class IIa histone deacetylases (HDACs) family, HDAC5 functions as a scaffolding protein that mediates the deacetylation of both histone and non-histone substrates 14, 15, thereby modulating oncogenic signaling pathways and implicating in tumor cell proliferation, metastasis, metabolism, immune response, and drug sensitivity 16-18. Loss of HDAC5 expression has been reported in multiple malignancies, and HDAC5 deficiency is associated with significantly reduced survival in patients with pancreatic ductal adenocarcinoma (PDAC) 15, 17. Recent studies have also indicated that HDAC5 plays an important role in DNA damage repair 18; however, the underlying mechanisms remain incompletely understood.

In this study, we delineate the mechanism by which CK2 and HDAC5 regulate DNA end resection and DSB repair pathway choice through the modulation of Ku70 acetylation in PDAC. Under DNA damage stress, CK2 facilitates the nuclear translocation of cytoplasmic HDAC5 by mediating its phosphorylation. Once in the nuclear, HDAC5 promotes the removal of Ku70 from DSB sites by deacetylating Ku70 at lysine 287 (K287), thereby enhancing DNA end resection and promoting HR. In contrast, CK2 inhibition or HDAC5 loss in PDAC results in hyperacetylation of Ku70 at K287 and prolonged retention of the Ku heterodimer at DSB sites, leading to impaired DNA end resection, suppresses HR, and increased sensitivity to PARP inhibitors.

## Methods

### Cell lines and cell culture

The human PANC-1 (RRID: CVCL_0480), MIA PaCa-2 (RRID: CVCL_0428), HEK293T (RRID: CVCL_0063), and U2OS (RRID: CVCL_0042) cell lines were purchased from Procell Life Science & Technology (Wuhan, China). The PANC-1, MIA PaCa-2 and HEK293T cell lines were cultured in Dulbecco's Modified Eagle Medium (DMEM, 11965092, Gibco, USA), supplemented with 10% Fetal Bovine Serum (FBS; 10099141, Gibco, USA). The U2OS cell lines were cultured in specific mediums (CM-0236, Procell). All cell lines were incubated at 37 ℃ in a humidified atmosphere containing 5% CO_2_. Routine screenings for mycoplasma contamination were conducted throughout the study period, with all cell lines consistently testing negative.

### High-throughput screening

PANC-1 cells were cultured in DMEM supplemented with 10% FBS and were digested to a single cell suspension using 1× TrypZean Solution (Sigma-Aldrich, USA). Then, cells were seeded at a density of 1,000 cells per well in a final volume of 100 μL per well in 96-well plates using a Multidrop®™ Combi Reagent Dispenser (Thermo Fisher Scientific). After overnight incubation at 37 ℃ in a humidified atmosphere containing 5% CO_2_, cells were treated with the compound library. All compounds were obtained from SelleckChem dissolved either in dimethylsulfoxide (DMSO) or double-deionized water, resulting in each drug being applied at 10 μM. Cell viability was measured after 48 h using CCK-8 assay.

### Transfection and lentiviral infection

Cells were transiently transfected with Lipofectamine 2000 (Thermo Fisher Scientific, USA). Viral supernatant was collected two times (24 h and 48 h) after the co-transfection of lentiviral vectors and packaging plasmids (pMDLg, VSV-g and Rev). Collected lentiviruses were added to the PANC-1, MIA PaCa-2, HEK293T, and U2OS cells for further experiments with 8 mg/mL polybrene which enhances infection efficiency. After 24 h infection, infected cells were selected with 10 μg/mL of puromycin.

### Generation of knock in cell lines

To generate Ku70 KR mutation cell lines, the guide RNAs were cloned into pSpCas9-2A-Puro (PX459) vector. PANC-1 cells were co-transfected with PX459 vectors and pMD19-T plasmids containing the KR point mutation as the template for homology repair. After selection using 2mg/mL puromycin for 36 h, the cells seeded into 96-well plates for generating monoclonal cell populations. Knock-in efficiency was validated by genomic sequence and immunoblotting. All gRNAs used in this study are listed in Supplementary Table S1.

### Western blot and immunoprecipitation

Cells were harvested and lysed by RIPA Lysis Buffer (Beyotime, China). The supernatant was quantified by BCA Protein quantification assay (Beyotime, China). The samples were subjected to SDS-PAGE gels and then transferred to PVDF membranes (Pierce Biotechnology, USA). Membranes were blocked for 1 h at room temperature with 5% skimmed milk and then incubated with primary antibodies at 4 ℃ overnight. The next day, the membranes were washed three times with 1× TBST, followed by secondary antibodies for 1 h at room temperature. Finally, after washing with 1× TBST for three times, the membranes were visualized by Bio-Rad Image Lab using ECL detection reagents (Thermo Fisher Scientific, USA). For immunoprecipitation, protein A/G agarose beads (Beyotime, China) and a primary antibody were co-cultured with protein on the rotary mixer over 24 h at 4°C. The beads were collected by centrifugation and washed for six times with PBS buffer. Beads were resuspended with 1× SDS-PAGD loading buffer and boiled for 10 min at 95 ℃, and subjected to Western blot analysis.

### GST pull-down assay

Bacterially expressed GST-fused HDAC5 was co-incubated with lysis buffer and GST-tagged protein purification agarose magnetic beads (Beyotime, China), and subsequent washing steps led to the purification of HDAC5. Recombination human Ku70 protein and recombination human Ku80 protein were purchased from Abcam (UK). Then, GST pull-down experiment was carried out with the GST Protein Interaction Pull-Down Kit (Thermo Scientific, USA).

### Cell proliferation assay

Cell proliferation capacity was assessed through the Cell Counting Kit-8 (CCK-8) assay. Amount count of 2,000 cells were resuspended in 200 μL of medium and cultured in 96-well plates for 5 days. The CCK-8 reagent (#C0037, Beyotime) was added to each well 1 h and the absorbance at 450 nm was measured with a microplate reader.

### Real-time PCR

Total RNA was extracted utilizing TRIzol reagent (Thermo Fisher Scientific, USA). The concentration and quality of RNA were then assessed using a Nano-Drop 2000 (Thermo Fisher Scientific, USA). RNA was reverse-transcribed with a Prime Script^TM^ RT Reagent Kit (Takara), according to the manufacturer's protocol. Real-time PCR was performed with TB Green^TM^ Fast qPCR Mix (Takara, Japan) under the following conditions: 95 ℃, 30 s; (95 ℃, 5 s; 60 ℃, 30 s) × 40. All primer sequences are provided in Supplementary Table S1. Relative gene expression levels were calculated using the 2-ΔCt method, with GAPDH mRNA levels serving as the reference.

### HR and NHEJ report assay

Cells were transfected with shNC, shHDAC5, shBRCA1 or sh53BP1, separately, and with combinations of HR (pDR-GFP)- or NHEJ (pPEM1-Ad2-EGFP)-reporter constructs and an expression vector for the restriction enzyme Ⅰ-Sce Ⅰ. All plasmids used in the GFP-reporter assay were a gift of Z. Lou (Mayo Clinic). PANC-1 cells integrated with a DR-GFP cassette as reported previously 19, 20 were used to analyze chromosomal HR efficiency. The GFP expression induced by the positive control plasmid was used to normalize the electroporation efficiency. Cells were grown for 48 h and processed for further flow cytometry analysis.

### Comet assay

Cells were seeded into 6-well plates with a density of 4,000 cells per well. After treatment with ionizing radiation (4 Gy), cells were harvested at the indicated time and then utilized for comet assays according to the manufacturer's instructions. Briefly, cells were embedded in low-melting agarose on glass slides and incubated in lysis buffer for 1 h at 4 ℃. The slides were incubated subsequently with pre-chilled alkaline solution for 30 min. Electrophoresis was then carried out at 25 V for 25 min. Next, the slides were stained with PI and observed using a fluorescence microscope. Tail DNA percent was measured to evaluate the degree of DNA damage.

### Karyotype analysis

Cells were treated with ionizing radiation (4 Gy) and then, 4 h later, arrested with colcemid (0.1 μg/mL) at 37 ℃ for 1 h before harvested, resuspended in pre-warmed hypotonic solution (0.075 M KCl) at 37 ℃ for about 30 min. After the samples were centrifuged (300 g, 5 min) and supernatant discarded, cells were fixed with fixative (3:1 methanol:glacial acetic acid) twice, for 8 min each time. Next, the cells were resuspended with fresh fixative and dropped onto slides, dried for about 30 min at about 65 ℃. Slides were stained with Diff-Quik (Solarbio, China) for 1 min. Genomic instability was analyzed by counting cells which showed chromosome breaks.

### Immunofluorescence and foci quantification

Cells were seeded into 12-well plates with coverslips (Solarbio, China) after treatment according to the experimental design. Cells were fixed with pre-chilled methanol at -20 ℃ for 30 min, and then washed twice using PBS. Subsequently, 600 μL of 0.5% Triton X-100 (Solarbio, China) was added to each well, standing 15 min at room temperature, and then washed twice using PBS. Next, cells were blocked with 5% Bovine serum albumin (BSA) (Beyotime, China) for 1 h at room temperature, and then washed twice using PBS. Cells were incubated with primary antibodies overnight at 4 ℃. After washing three times with PBS, cells were incubated with 1 mL prepared FITC-goat anti-rabbit/mouse IgG (1:1000 dilution for ICC, Abcam) to each well at dark environment. After washing three times with PBS, cells were incubated with DAPI staining solution (Beyotime, China) at room temperature for about 15 min. After washing them twice with PBS, the coverslips added anti-fluorescence decay mounting medium (Beyotime, China) were observe under a confocal micro-scope. Depending on the experiment, DNA damage foci were counted manually, count at least 50 cells per group.

### Detection of end resection using qPCR

AsiSI-ER U2OS cells which were advance-infected as indicated were treated with 300 nM 4-Hydroxytamoxifen (4-OHT) (Sigma-Aldrich, USA) for 4 h. Genomic DNA was extracted. The genomic DNA sample was digested with restriction enzyme BamHI (Thermo Fisher Scientific, USA) at 37 ℃ overnight. The extent of resection was determined by qPCR with TaqMan and primer pairs for AsiSI site located on chromosome 1 (DSB, Chr 1: 109838221). In addition, a primer pair across a HindIII restriction site on chromosome 22 with no DSB is used as negative control. The primer sequences are provided in Table S1. The percentage of single-stranded DNA (ssDNA%) generated by resection at selected sites was calculated as: ssDNA% = 1/(2(ΔCt - 1) + 0.5) ×100. where ΔCt is obtained by subtracting the Ct value of the untreated sample from the Ct value of the 4-OHT-treated sample.

### Colony formation assay

The indicated cells were seeded in triplicate in 6-well plates (1000 cells per well). Cells were treated with olaparib (S1060, Selleck), silmitasertib (S2248, Selleck), or both, and left for 14 days in the incubator to allow colony formation. Then, the medium was removed and cells were fixed by 4% paraformaldehyde (Beyotime, China) for 30 min. Colonies were stained with Crystal Violet Staining Solution (Beyotime, China) and quantified.

### 3D (three-dimensional) culture

Thaw the Matrigel matrix (Corning, USA) overnight at 4 °C in the refrigerator before use. Add 200 μL of Matrigel matrix into each well of a pre-chilled 24-well plate, spread evenly with a pipet tip, and then incubate at 37 ℃ for 30 min, to allow the Matrigel matrix to gel. Plate 250 μL prepared cell suspension into each well of the pre-coated 24-well plate, and incubate at 37 ℃ for 30 min. Chill the DMEM complete medium and add Matrigel matrix to 10% of the final volume. Gently add 250 μL of Matrigel matrix medium mixture to the plated culture. Continuously culture for 7 days and observe cell morphology, and the Matrigel matrix medium mixture was changed every 2 days.

### Generation of the xenograft pancreatic cancer mouse model

BALB/c nude mice (5 weeks old, male) were purchased from Vitalriver (Beijing, China). All mice were housed in standard conditions (5 mice per cage) and provided free access to diet and water. Mice were randomized to experimental arms prior to cell implantation and/or treatment. PANC-1 cells infected with different lentivirus were resuspend in 100 μL PBS and subcutaneously injected into the left back of nude mice (5 × 10^6^ cells per mice). And the mice were administered olaparib (orally, 50 mg/kg) (S1060, Selleck) or vehicle every day. The size of heterografts was monitored every three days and tumor volume was calculated using the formula: (Length × Width^2^)/2. At the end point of the experiment, the mice were euthanized and the tumors were excised and weighed. Animal experiments were conducted in accordance with ethical guidelines and approved by the Institutional Animal Care and Use Committee (IACUC) of Tongji Medical College, Huazhong University of Science and Technology (Approval No. IACUC 4405).

For assays testing the combination of silmitasertib and olaparib to kill pancreatic cancer, PANC-1 cells were resuspended in 100μl PBS and subcutaneously injected into the left back of nude mice (5 × 10^6^ cells per mice). Tumor-bearing mice were randomly grouped when the average tumor volume reached approximately 100 mm^3^. Mice were randomized into 4 groups of 6 mice: 1) group 1: control; 2) group 2: treated with silmitasertib (S2248, Selleck); 3) group 3: treated with olaparib (S1060, Selleck); 4) group 4: treated with silmitasertib and olaparib. All drugs were administered via oral gavages at different concentration: silmitasertib, 75mg/kg twice daily for 3 weeks; olaparib, 50 mg/kg once daily for 3 weeks. The size of heterografts was monitored every three days and tumor volume was calculated using the formula: (Length × Width^2^)/2. At the end point of the experiment, the mice were euthanized and the tumors were excised and weighed. All experimental procedures with mice were conducted per the guidelines approved by the local ethics committee (Tongji Medical College, HUST, China).

### Patient-derived organoid models

Organoids were initiated using surgically resected PDAC tissue obtained from the primary tumor with pathologic evaluation. Six pancreatic cancer organoids in good condition were passed and seeded in standard 96-well cell culture plates with a scalpel and enzymatic dissociation in a digestion media comprising culture medium and collagenase Ⅺ (Cat#C9407, Sigma-Aldrich). The resulting single cells were suspended in liquid Matrigel, plated in domes, and covered with human complete feeding media. Cultures underwent biweekly passaging for cell line expansion or characterization, and the size of the organoids was measured on a weekly basis.

### Spontaneous pancreatic cancer model

*Hadc5*-knockout or wild-type KPC transgenic mice (*SL-KrasG12D/+; LSL-Trp53R172H/+; Pdx-1-Cre;* 8-week-old, sex-matched) were purchased from Cyagen (Suzhou, China) and housed under pathogen-free conditions. And the mice were administered vehicle, olaparib (50 mg/kg), silmitasertib (75 mg/kg) or both of them. All drugs were administered via oral gavage at different concentration: silmitasertib, twice daily; olaparib, once daily. Mice were euthanized, and the pancreas was collected after 5 weeks.

### Tissue microarray and immunohistochemistry (IHC)

The tissue microarray (TMA) slides were purchased from Avilabio (Shanxi, China) (DC-Pan01020). The TMA slides were immunostained with respective specific antibodies. The staining intensity was scored in blinded fashion: 1 = weak staining; 2 = medium staining; 3 = strong staining. The positive percentage was defined as follow: 0=0%, 1=1-25%, 2=26-50%, 3=51-75%, 4=above 75%. The staining intensity was calculated by the function: SI = (positive cells% * the staining intensity). Then, the median value of IHC scoring in all samples was chosen as the cut-off value. Two independent pathologists who were not aware of the experiments performed the assessment. The information of antibodies is provided in Key Resource Table. The IHC was conducted at BIOSSCI Biotech Co., Ltd (Hubei, China).

### Quantification and statistical analysis

Data analysis was carried out using GraphPad Prism 9. Statistical significance was calculated by unpaired two-tailed Student's t-test between two groups or by one-way or two-way ANOVA with Tukey's corrections when comparing three or more groups. Data are presented as mean ± standard deviation (SD). Each independent experiment was conducted with biologically independent experiments. *P* values under 0.05 were considered as statistically significant for all tests.

## Results

### HDAC5 loss induces sensitization to PARP inhibitors in homologous recombination-proficient PDAC

**Figure 1:**
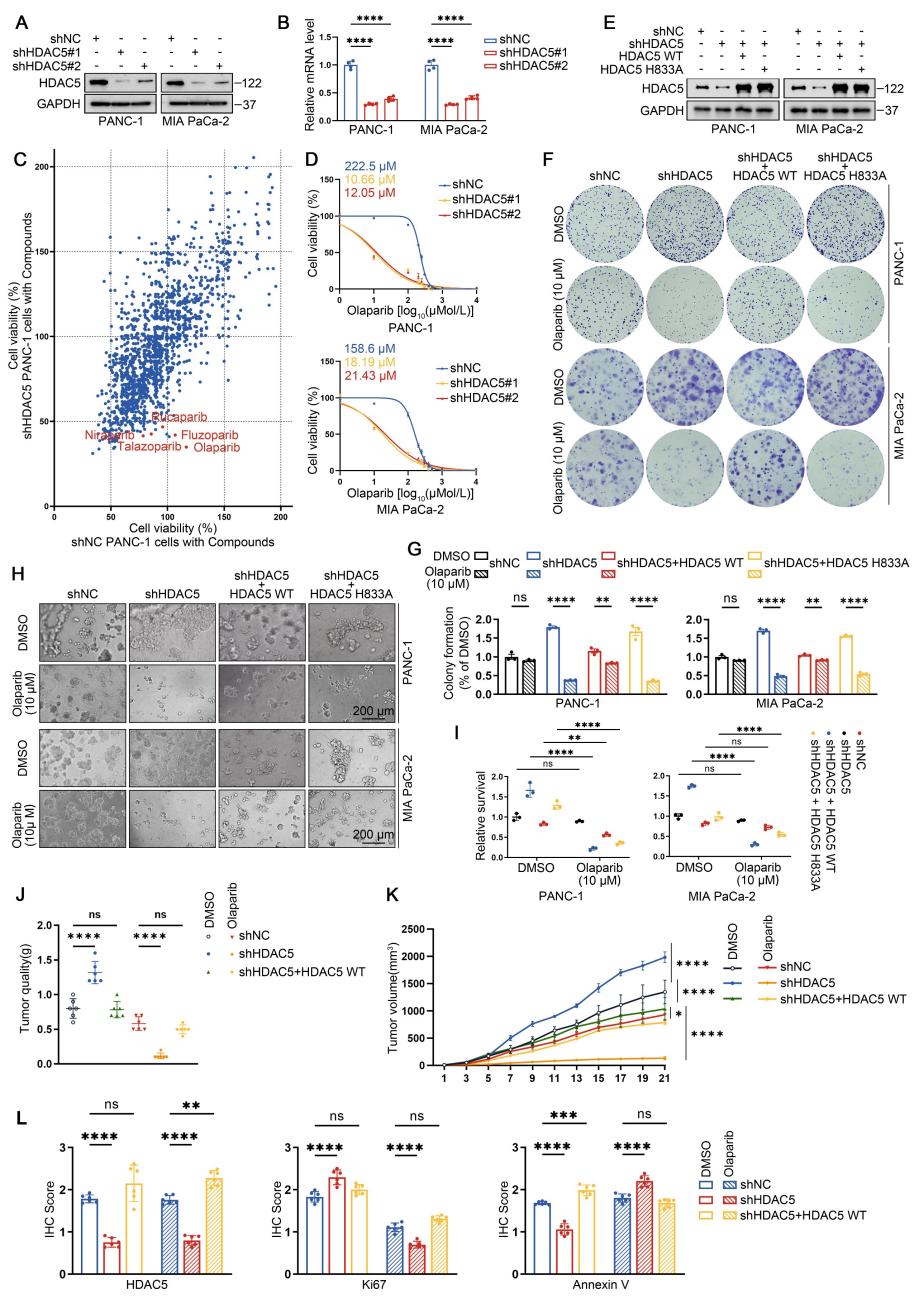
** HDAC5 loss induces sensitization to PARP inhibitors in homologous recombination proficient PDAC. (A)** Western blots show the knockdown efficiency of HDAC5 in PANC-1 and MIA PaCa-2 cells. shNC denotes non-targeting shRNA control. **(B)** RT-qPCR analysis of HDAC5 mRNA expression in PANC-1 and MIA PaCa-2 cells. Data are shown as mean ± SD (n=4 technical replicates). P values were derived from a two-way ANOVA. **(C)** Scatter plot presents the cell viability results at 48 h post treatment for a screen of 1734 compounds (10 μM) targeting PANC-1 cells transfected with either shNC (on the x-axis) or shHDAC5 (on the y-axis). Each point represents an individual data point, with the position of the point determined by the values of PANC-1 cells transfected with either shNC or shHDAC5 for that specific data point. **(D)** Dose-response survival curves of shNC, shHDAC5#1, and shHDAC5#2 expressing cells exposed to escalating concentrations of olaparib in both PANC-1 (top panel) and MIA PaCa-2 (bottom panel) cell lines. Cell viability was assessed at the end of the 5-day treatment period. Data are shown as mean ± SD (n=3 biological replicates). **(E)** Western blots of HDAC5 expression in PANC-1 and MIA PaCa-2 cells. **(F-G)** Colony formation assays were conducted in PANC-1 (top) and Mia PaCa-2 (bottom) cells. The cell lines were transfected as indicated and subsequently treated with DMSO or the PARP inhibitor olaparib at a concentration of 10 μM. Cells were allowed to grow for 14 days before staining. Representative colonies images are shown in **(F)**, with quantification data**(G)**. Data are shown as mean ± SD (n=3 biological replicates). P values were derived from a two-way ANOVA. **(H-I)** 3D cultures were conducted in PANC-1 (top) and Mia PaCa-2 (bottom) cells. Cells were transfected as indicated and subsequently treated with DMSO or the olaparib (10 μM), with samples collected and analyzed following a 7-day treatment period. Scale bar, 200 μm. Representative images are shown in **(H)**, with quantification data **(I)**. Data are shown as mean ± SD (n=3 biological replicates). P values were derived from a two-way ANOVA. **(J-K)** Tumor quality was measured **(J)**; and volumes were calculated **(K)**. Data were shown as mean ± SD (n=6 biological replicates). P values were derived from the one-way ANOVA (tumor quality) and the two-way ANOVA (tumor volume). **(L)** IHC scores of IHC staining in Supplementary Figure 1C of HDAC5, Ki67, and Annexin Ⅴ form xenograft tumors. P values were derived from a one-way ANOVA. See also Supplementary Figure 1.

To further investigate the role of HDAC5 in PDAC and explore potential therapeutic vulnerabilities associated with HDAC5 deficiency, we generated stable HDAC5 knockdown cell lines in two human PDAC cell lines using two independent shRNAs (Figure 1A-B). Using an anticancer compound library, we screened control and HDAC5-depleted PANC-1 cells and observed a marked increase in sensitivity to several PARP inhibitors upon HDAC5 knockdown (Figure 1C). Dose-response analyses in PANC-1 and MIA PaCa-2 cells showed that HDAC5 deficiency resulted in a pronounces downward shift of olaparib dose-response curves compared with control cells. Accordingly, the IC50 values decreased from 222.5 μM to 10.66 μM and 12.05 μM in PANC-1 cells, and from 158.6 μM to 18.19 μM and 21.43 μM in MIA PaCa-2 cells (Figure 1D). Similar sensitization effects were confirmed using additional PARP inhibitors, including niraparib, rucaparib and talazoparib (Figure S1A). To determine whether this effect depends on HDAC5 enzymatic activity, we re-expressed wild-type (WT) HDAC5 or an enzymatically inactive mutant (H833A) in HDAC5-depleted PANC-1 and MIA PaCa-2 cells (Figure 1E). Notably, only re-expression of WT HDAC5 restored resistance to PARP inhibition, whereas re-expression of the catalytically inactive HDAC5 mutant had no detectable effect, as assessed by both colony formation and 3D culture assays (Figure 1F-I). We further validated these observations using a subcutaneous xenograft model with patient-derived PDAC cells (PDCs). Olaparib treatment robustly suppressed tumor growth of HDAC5-deficient xenografts, whereas no comparable inhibitory effect was observed in control tumors or in tumors with HDAC5 re-expression (Figure 1J-L and Figure S1B-C). Collectively, these results indicate that loss of HDAC5 sensitizes PDAC models to PARP inhibition.

### HDAC5 maintains HR function in an enzyme-dependent manner

**Figure 2:**
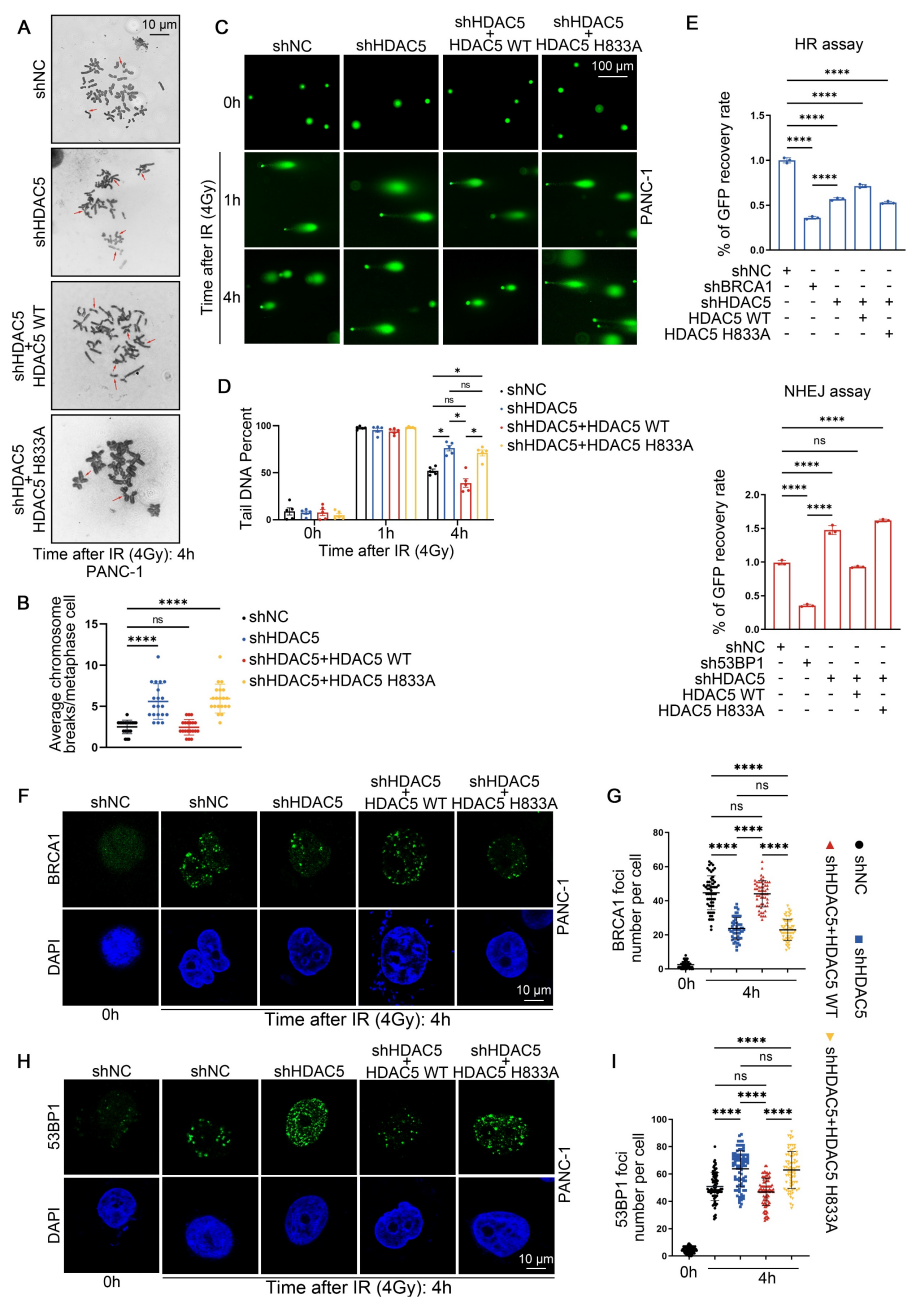
** HDAC5 maintains HR function in an enzyme-dependent manner. (A-B)** Representative karyotypic images showing metaphase chromosome spreads in PANC-1 cells transfected as indicated, for the purpose of quantifying chromosomal aberrations. **(A)** Representative aberrations are marked by arrows. Scale bar, 10 μm. **(B)** The average results from three independent experiments with a minimum of 20 cells counted in each experiment. Data were shown as mean ± SD. P values were derived from a one-way ANOVA. **(C-D)** Representative images from comet assays performed under neutral conditions are shown at 1 h and 4 h post-treatment with 4 Gy irradiation, showing delayed repair of DNA in PANC-1 cells transfected as indicated. **(C)** Representative images show the migration of DNA fragments (comet tail) after irradiation. Scale bar, 100 μm. **(D)** Comet tail DNA precent were quantified from one of three biological replicated (n=5 technical replicates). Data were shown as mean ± SD. P values were derived from a one-way ANOVA.** (E)** Characterization of Double-stranded DNA breaks repair pathway by pDR-GFP (top panel) and pPEM1-Ad2-EGFP (bottom panel). Quantification of GFP^+^ PANC-1 cells was analyzed by flow cytometry. BRCA1 and 53BP1 are positive controls. Data were shown as mean ± SD (n=3 biological replicates). P values were derived from a one-way ANOVA. **(F-I)** Representative fluorescence images of BRCA1**(F)** and 53BP1 **(H)** foci in as indicated PANC-1 cells. Scale bar, 10 μm. Quantification of the average number of BRCA1 **(G)** and 53BP1 **(I)** foci per focus-positive cell. Data were shown as mean ± SD of more than 50 cells. P values were derived from a two-way ANOVA. See also Supplementary Figure 2-3.

**Figure 3:**
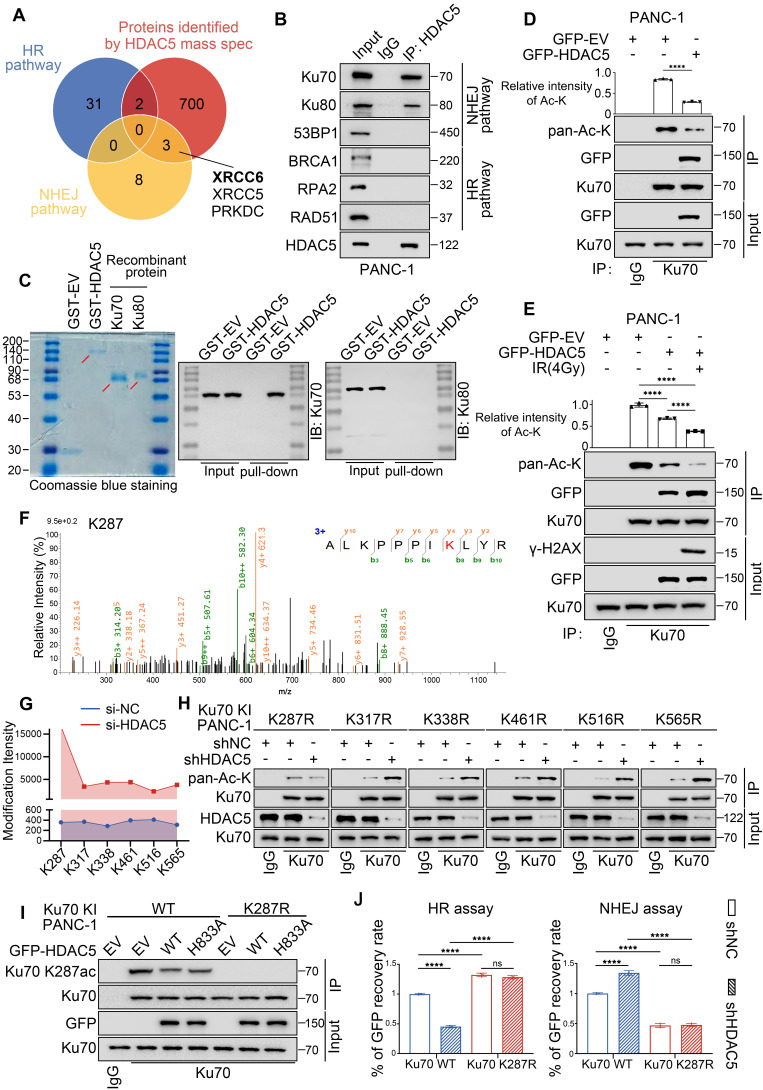
** HDAC5 regulates DSB repair through site-specific deacetylation of Ku70 at K287. (A)** Venn diagram shows the overlap of related genes in HR pathway, related genes in NHEJ pathway and mass spectrometry data of protein samples purified by HDAC5 specific antibody. XRCC6 and XRCC5 genes encode Ku70 and Ku80, respectively, which play a major role in the NHEJ pathway. **(B)** The interaction between HDAC5 and HR/NHEJ pathway pivotal protein in PANC-1 cells. **(C)** Coomassie Brilliant Blue staining of purified GST-HDAC5 fusion protein, recombinant Ku70 protein and recombinant Ku80 protein (left panel). The GST pull-down assays were performed by incubating GST or GST-HDAC5 with recombinant Ku70 or Ku80 separately. The pulled-down complexes were immunoblotted with the indicated antibodies to detect associated Ku70 or Ku80. **(D)** Decreased HDAC5-induced Ku70 acetylation upon HDAC5 overexpression in PANC-1 cells. Relative intensities of acetylation lysine are mean ± SD of three independent experiments. P values were derived from a one-way ANOVA. **(E)** Decreased HDAC5-induced Ku70 acetylation upon 1 h post-treatment with 4 Gy irradiation in PANC-1 cells. Relative intensities of acetylation lysine are mean ± SD of three independent experiments. P values were derived from a two-way ANOVA. **(F)** Illustration of Ku70 acetylation at K287 identified by mass spectrometry. **(G)** Line chart showing the intensities of Ku70 acetylation at K287, K317, K338, K461, K516 and K565 identified by mass spectrometry in Si-NC and Si-HDAC5 groups. **(H)** Western blot analysis of Ku70 acetylation. Increased Ku70 acetylation upon HDAC5 depletion in Ku70 knock-in PANC-1 cells with mutations at K317, K338, K461, K516 and K565. Ku70 acetylation remains unaffected by HDAC5 depletion in Ku70 knock-in PANC-1 cells with mutations at K287. **(I)** Western blot analysis of Ku70 K287 acetylation in Ku70 knock-in PANC-1 cells.** (J)** Flow cytometry analyzed the percentage of GFP^+^ Ku70 knock-in PANC-1cells. Data are shown as mean ± SD. P values were derived from a two-way ANOVA. See also Supplementary Figure 4.

We next examined whether HDAC5 contributes to DNA damage repair and the maintenance of genome stability. To induce DNA damage, PANC-1 and MIA PaCa-2 cells were exposed to 4 Gy of ionizing radiation (IR). Karyotype analysis revealed that, 4 h after IR, chromosome breaks were significantly increased in HDAC5-depleted cells compared with control cells (Figure 2A-B and Figure S2A-B). This phenotype was rescued by re-expression of WT HDAC5, but not by the enzymatically inactivate HDAC5 mutant (H833A) (Figure 2A-B and Figure S2A-B). We next performed single-cell comet assays to assess DNA damage and repair dynamics. One h after IR exposure, comparable levels of DNA damage were detected across all experimental groups in both PANC-1 and MIA PaCa-2 cells, irrespective of HDAC5 status (Figure 2C and Figure S2C). Consistently, quantitative analysis of comet tail DNA revealed no significant differences among the groups at this early time point (Figure 3D and Figure S3D). By 4 h after IR treatment, comet tails in control cells and in cells re-expressing WT HDAC5 were shortened by approximately 50%, consistent with efficient DNA repair. In contrast, HDAC5 knockdown cells and cells expressing the enzymatically inactive HDAC5 mutant (H833A) showed markedly delayed tail shortening, with reductions of only ~20% (Figure 3C-D and Figure S3C-D). These results indicate that loss of HDAC5 compromises DNA damage repair and promotes chromosomal instability in pancreatic cancer cells.

Given the pronounced effect of HDAC5 on PARP inhibitor sensitivity, we next investigated whether HDAC5 directly influences DSB repair pathway activity. HR reporter assays showed that HDAC5 knockdown markedly suppressed HR, an effect that was rescued by re-expression of WT HDAC5, but not by the the enzymatically inactive HDAC5 mutant (H833A) (Figure 2E). Conversely, HDAC5 depletion led to increased NHEJ activity, which was similarly reversed by re-expression of WT HDAC5 (Figure 2E). We next assessed ionizing radiation-induced foci (IRIF) formation of BRCA1 and 53BP1, key mediators of HR and NHEJ, respectively. Consistent with the reporter assay results, HDAC5 knockdown resulted in a reduced number of BRCA1 foci and a concomitant increase in 53BP1 foci in both PDAC cell lines at 4 h after IR (Figure 2F-I and Figure S2E-H). Importantly, these alterations were reversed only by the re-expression of enzymatically competent WT HDAC5 (Figure 2F-I and Figure S2E-H). Collectively, these data indicate that HDAC5 maintains HR function in an enzyme-dependent manner.

### HDAC5 regulates DSB repair through site-specific deacetylation of Ku70 at K287

HDAC5 is well recognized as a transcriptional regulator. To determine whether HDAC5 influences DSB repair through transcriptional mechanisms, we analyzed RNA-seq data from control and HDAC5 knockdown PANC-1 cells. Notably, HDAC5 depletion did not lead to significant changes in the mRNA expression of genes involved in DSB repair (Figure S3A-B). Consistent with these findings, RT-qPCR analysis of representative HR- and NHEJ-related genes yielded similar results (Figure S3C). Furthermore, western blot (WB) analysis confirmed that HDAC5 knockdown did not alter the protein expression levels of these factors, either in the presence or absence of IR (Figure S3D). Given that HDAC5-mediated regulation of DSB repair depends on its enzymatic activity, yet does not involve changes in the expression of canonical repair factors, we reasoned that HDAC5 may regulate DSB repair through deacetylation of non-histone DNA repair proteins. Supporting this possibility, mass spectrometry (MS) analysis identified Ku70 and Ku80 as prominent HDAC5-interacting proteins (Figure 3A). Reciprocal co-immunoprecipitation (Co-IP) assays further confirmed endogenous interactions between HDAC5 and the Ku70/Ku80 complex (Figure 3B and Figure S4A). However, in vitro GST pull-down assays revealed that GST-tagged HDAC5 directly interacted with recombinant Ku70, but not with recombinant Ku80. These results indicate that HDAC5 specifically associates with Ku70, whereas its interaction with Ku80 in vivo is likely indirect and mediated through the pre-assembled Ku heterodimer (Figure 3C).

Consistent with the GST pull-down results, overexpression of WT HDAC5 selectively reduced the acetylation level of Ku70, while having no detectable effect on Ku80 acetylation (Figure 3D and Figure S4B-C). Notably, IR treatment markedly enhanced the interaction between HDAC5 and Ku70, which was accompanied by a further reduction in Ku70 acetylation (Figure 3E). These findings suggest that HDAC5 deacetylates Ku70 in response to DNA damage.

To further define the region of Ku70 responsible for HDAC5 interaction and regulation, we generated a series of Ku70 truncation constructs encompassing three domains: the N-terminal fragment (residues 1-260) containing the vWA domain, the central DNA-binding domain (residues 261-480), and the C-terminal region (residues 481-609) harboring the flexible linker as well as the recently described nSAP and cSAP subdomains 21 (Figure S4D). Co-IP analysis demonstrated that HDAC5 interacted with both the 1-260 and 261-480 fragments of Ku70 (Figure S4E), indicating that the interaction likely involves a conformational or extended interface rather than a discrete linear sequence. To pinpoint the specific lysine residue on Ku70 regulated by HDAC5, we performed quantitative MS analysis of Ku70 acetylation in control and HDAC5 knockdown PANC-1 cells. This analysis identified six lysine residues—K287, K317, K338, K461, K516, and K565—whose acetylation levels were increased upon HDAC5 depletion (Figure 3F and Figure S4F). Among these sites, K287, located within the 261-480 DNA-binding region of Ku70, exhibited the most pronounced increase in acetylation intensity (15697.34614/359.071333) (Figure 3G). To functionally validate these findings, we generated acetylation-resistant Ku70 mutants by individually substituting each of the six lysine sites with arginine (K-to-R mutations). Co-IP analysis demonstrated that only the Ku70-K287R mutation abolished the increase in Ku70 acetylation induced by HDAC5 knockdown, whereas mutations at the other lysine sites had no such effect (Figure 3H). To further validate this hypothesis, we generated WT or K287R knock-in PANC-1 cells, and Co-IP analysis indicated that Ku70 K287R mutant significantly downregulated Ku70 acetylation (Figure S4G). Moreover, ectopic overexpression of WT HDAC5 repressed the acetylation of WT-Ku70 but had no effect on the acetylation level of the Ku70-K287R mutant (Figure S4G). We further generated an antibody specifically recognizing K287 acetylated Ku70 (Figure S4H), and repeated the Co-IP assays, which yield consistent results (Figure 3I). Consistently, Ku70-K287R knock-in PANC-1 cells exhibited significantly enhanced HR activity and reduced NHEJ activity compared with WT Ku70 knock-in cells, and these effects were not further altered by HDAC5 knockdown (Figure 3J). Collectively, these data indicate that HDAC5 specifically regulates DNA double-strand break repair through deacetylation of Ku70 at lysine 287.

### HDAC5 deficiency-induced Ku70 K287 acetylation suppresses DNA end resection by prolonging Ku70 retention at DSB sites

**Figure 4:**
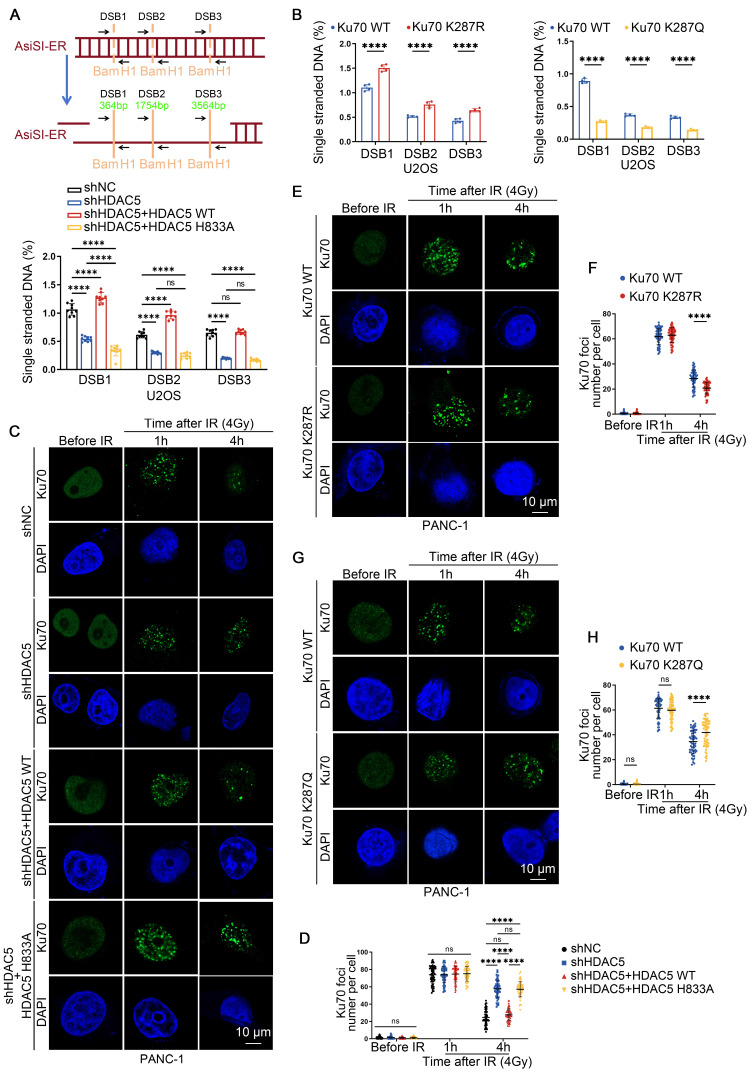
** HDAC5 deficiency-induced Ku70 K287 acetylation suppresses DNA end resection by prolonging Ku70 retention at DSB sites. (A)** AsiSI-ER U2OS cells transfected as indicated by lentivirus and treated with 300 nM 4-OHT for 4 h or mock-treated (top panel); DNA end resection adjacent to DSB1, DSB2, and DSB3 sites were measured by RT-qPCR as described in the Methods (bottom panel). The relative amount of PCR product from the +Enzyme sample, normalized to its No Enzyme control, quantitatively represents the proportion of ssDNA at that specific genomic location. A higher normalized value indicates more extensive resection. Data are shown as mean ± SD. P values were derived from a two-way ANOVA. **(B)** RT-qPCR analysis of detecting DNA end resection in AsiSI-ER U2OS cells transfected with plasmids as indicated. Data are shown as mean ± SD. P values were derived from a two-way ANOVA. **(C-H)** Representative fluorescence images of Ku70 foci **(C, E, G)** in as indicated PANC-1 cells. Scale bar, 10 μm. Quantification of the average number of Ku70 foci **(D, F, H)** per focus-positive cell. Data were shown as mean ± SD of more than 50 cells. P values were derived from a two-way ANOVA. See also Supplementary Figure 5-8.

The Ku70/80 heterodimer rapidly occupies the DSB sites following damage, thereby inhibiting DNA end resection and influencing the choice between HR and NHEJ pathways 22-24. Given that HDAC5 modulates the HR/NHEJ pathway balance through deacetylation of Ku70 at K287, we next asked whether Ku70 K287 acetylation affects Ku70 retention at DSB sites and the efficiency of DNA end resection. We first assessed DNA end resection using a qPCR-based in U2OS cells 25. HDAC5 knockdown led to a significant reduction in the proportion of ssDNA, indicating marked suppression of DNA end resection. This defect was rescued by re-expression of WT HDAC5, but not by the enzymatically inactive HDAC5 mutant (H833A) (Figure 4A). Consistent with these observations, Ku70-K287R knock-in PANC-1 cells exhibited a significant increase in DNA end resection compared with WT Ku70 knock-in cells. In contrast, knock-in of the acetylation-mimetic Ku70-K287Q mutant resulted in reduced DNA end resection activity (Figure 4B). Both in vitro electrophoretic mobility shift assays (EMSA) and cellular ChIP-qPCR analyses showed that the Ku70-K287Q mutant displayed enhanced DNA-binding affinity and prolonged retention at DSB sites. These results indicate that acetylation at K287 stabilizes the Ku70-DNA interaction, whereas deacetylation promotes Ku70 dissociation from DNA ends (Figure S7D-E).

Consistent with impaired DNA end resection, the numbers of IRIF positive for 5-bromo-2'-deoxyuridine (BrdU), RPA2 and RAD51 were significantly reduced in HDAC5 knockdown PANC-1 and MIA PaCa-2 cells. This reduction was rescued by re-expression of WT HDAC5, but not by the enzymatically inactive HDAC5 mutant (H833A) (Figure S5A-D). Similarly, Ku70-K287R knock-in PANC-1 cells showed increased BrdU, RPA2, RAD51 and BRCA1 foci compared with WT Ku70 knock-in PANC-1 cells, whereas Ku70-K287Q knock-in PANC-1 cells displayed the opposite phenotype (Figure S6A-J). Importantly, HDAC5 knockdown fail to further reduce resection-associated foci in Ku70-K287R knock-in cells, indicating that K287 deacetylation functions downstream of HDAC5 in regulating DNA end resection (Figure S7A-C). Analysis of Ku70 foci dynamics in PANC-1 and MIA PaCa-2 cells showed that HDAC5 depletion did not significantly affect initial Ku70 foci formation at 1 h after IR (Figure 4C-D and Figure S8A). In contrast, at 4 h after IR, HDAC5 depletion markedly prolonged Ku70 retention at DSB sites compared with control cells (Figure 4C-D and Figure S8A). Consistently, Ku70-K287R knock-in PANC-1 cells exhibited reduced Ku70 foci at 4 h after IR compared with WT Ku70 knock-in cells, whereas Ku70-K287Q knock-in PANC-1 cells exhibited prolonged Ku70 retention at DSB sites (Figure 4E-H). Moreover, HDAC5 knockdown failed to further enhance Ku70 retention in Ku70-K287R knock-in PANC-1 cells (Figure S8B). Together, these data indicated that HDAC5 loss promotes Ku70 retention at DSB sites and suppresses DNA end resection through regulation of Ku70 K287 acetylation.

### CK2 promotes DNA damage-induced nuclear translocation of HDAC5

**Figure 5:**
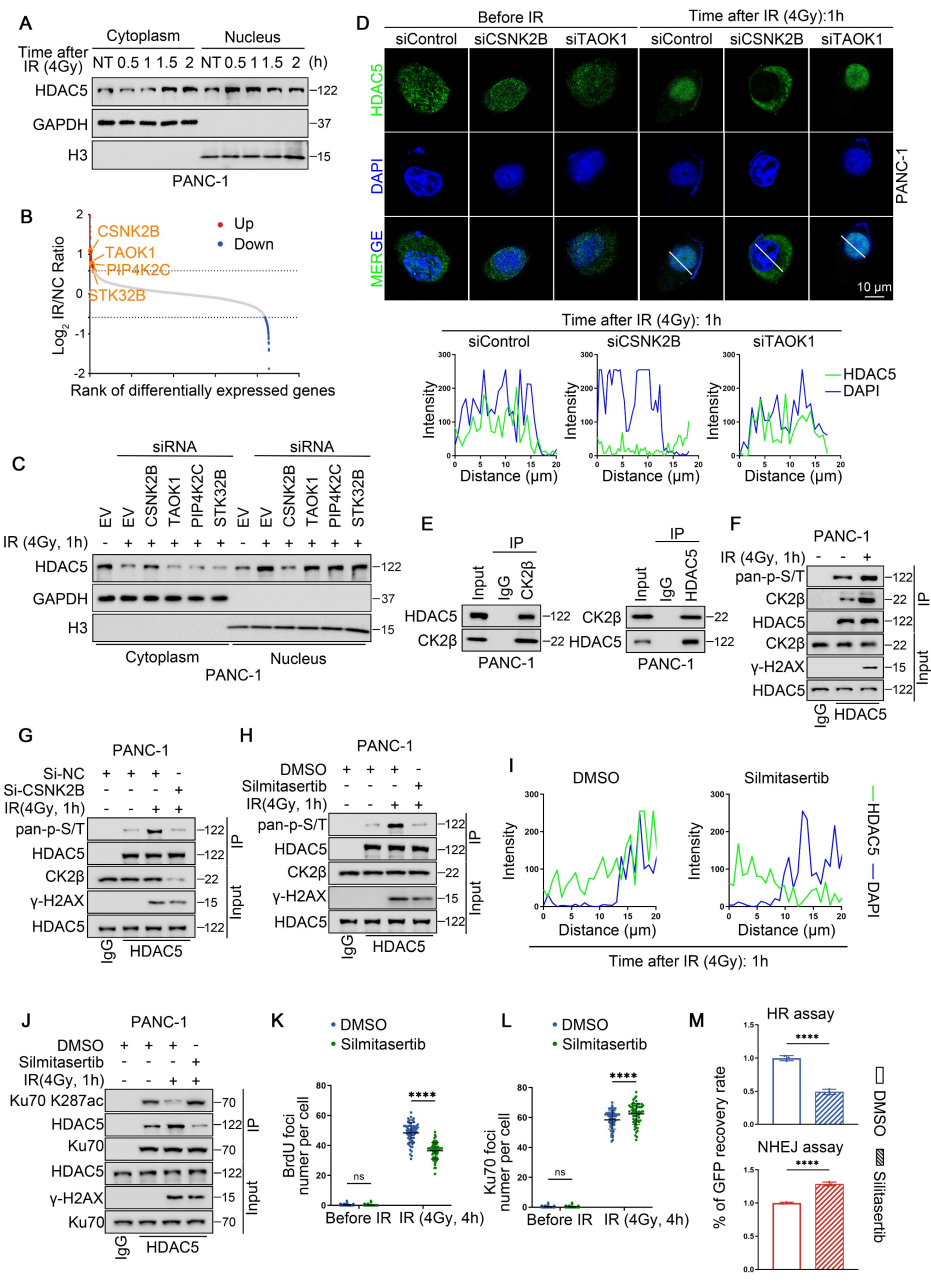
** CK2 promotes DNA damage-induced nuclear translocation of HDAC5. (A)** Western blot analysis of HDAC5 expression in both the nuclear and cytoplasmic fractions of PANC-1 cells treated with different time gradients after 4 Gy irradiation. **(B)** Scatter plot showing the differentially expressed genes identified by mass spectrometry purified by HDAC5 specific antibody in the control and irradiation group (4 Gy, 1 h). CSNK2B, TAOK1, PIP4K2C and STK32B are the genes encoding kinase-related proteins among the upregulated genes. **(C)** Western blot analysis of HDAC5 expression 1 h post-treatment with 4 Gy irradiation in both the nuclear and cytoplasmic fractions of PANC-1 cells transfected with siRNA as indicated. **(D)** Representative images of HDAC5 signals (green) in PANC-1 cells transfected with siRNA as indicated in the 1 h post-treatment with 4 irradiations (top panel). DAPI, nucleus. SiTAOK1, postive control. Scale bar, 10 μm. Plots of pixel intensity along the white line from left to the right of each plot (bottom panel), colors as in merged images.** (E)** The interaction between endogenous HDAC5 and CK2β in PANC-1 cells. **(F)** Increased CK2-induced HDAC5 phosphorylation upon 1 h post-treatment 4 Gy irradiation. **(G)** Decreased CK2-induced HDAC5 phosphorylation upon 1 h post-treatment 4 Gy irradiation and CSNK2B depletion. **(H)** Decreased CK2-induced HDAC5 phosphorylation was observed after 48-h pre-treatment with CK2 inhibitor silmitasertib (5 μM), followed by 1 h post-treatment 4 Gy irradiation.** (I)** Plots of pixel intensity along the white line from left to the right of each plot of the Supplementary Figure 9C, colors as in merged images. **(J)** Increased Ku70 K287 acetylation was observed after 48 h pre-treatment with CK2 inhibitor silmitasertib (5 μM), followed by 1 h post-treatment 4 Gy irradiation.** (K-L)** Quantification of the BrdU **(K)** and Ku70 **(L)** foci number per focus-positive cell in Supplementary Figure 9F-G. Data were shown as mean ± SD of more than 50 cells. P values were derived from a two-way ANOVA. **(M)** Flow cytometry analysis of the percentage of GFP^+^ PANC-1cells following treatment with DMSO or silmitasertib (5 μM) for 48 h. Data were shown as mean ± SD. P values were derived from the unpaired t test. See also Supplementary Figure 9.

HDAC5 is not traditionally regarded as a core DNA damage repair protein. However, IR markedly enhances its interaction with Ku70 and promotes Ku70 deacetylation (Figure 3E). Given that HDAC5 is a known nucleocytoplasmic shuttling protein, we next investigated whether DNA damage alters the intracellular localization of HDAC5, thereby facilitating its interaction with Ku70 26. Immunocytochemistry (ICC) revealed a pronounced increase in nuclear HDAC5 localization within 0.5-1 h following IR treatment (Figure S9A). Notably, both ICC and WB analyses showed that this nuclear accumulation was transient and largely diminished by 1.5 h after IR exposure (Figure 5A and Figure S9A-B). These observations suggest that HDAC5, similar to Ku70, functions as an early responder during the DSB repair process. To identify upstream kinases responsible for DNA damage-induced HDAC5 translocation, we performed MS analysis and identified several kinases whose interaction with HDAC5 was enhanced following IR treatment, including CSNK2B (CK2β), TAOK1, PIP4K2C and STK32B (Figure 5B). Subsequent siRNA-based screening revealed that depletion of CK2β, but not other candidate kinases, markedly suppressed IR-induced nuclear accumulation of HDAC5 (Figure 5C). This effect was further confirmed by ICC analysis (Figure 5D).

Reciprocal Co-IP assays in PANC-1 cells confirmed an endogenous interaction between CK2β and HDAC5 (Figure 5E). Consistent with this interaction, IR treatment enhanced CK2β-HDAC5 association and was accompanied by increased phosphorylation of HDAC5 (Figure 5F). In contrast, CK2β knockdown abolished the IR-induced increase in HDAC5 phosphorylation (Figure 5G). Treatment with silmitasertib, a selective CK2 inhibitor, significantly suppressed IR-induced HDAC5 phosphorylation (Figure 5H) and concomitantly reduced HDAC5 nuclear localization following DNA damage (Figure 5I and Figure S9C-D). As a consequence, silmitasertib treatment increased Ku70 K287 acetylation despite the presence of HDAC5, likely by preventing HDAC5 nuclear access and disrupting the HDAC5-Ku70 interaction (Figure 5J). Consistent with impaired DNA end resection, silmitasertib treatment reduced IR-induced ssDNA formation in U2OS cells (Figure S9E) and significantly decreased BrdU-positive foci at 4 h after IR in PANC-1 cells (Figure 5K and Figure S9F). In parallel, silmitasertib treatment resulted in increased Ku70 foci retention at DSB sites at the same time point (Figure 5L and Figure S9G). Accordingly, functional reporter assays demonstrated that CK2 inhibition suppressed HR activity while enhancing NHEJ activity (Figure 5M).

To identify the phosphorylation sites responsible for CK2-mediated regulation, we performed phosphoproteomic analysis and identified T552 and T577 as major IR-induced phosphorylation sites on HDAC5 (Figure S9H). Using HDAC5 knock-in PANC-1 cells, we found that phosphorylation at T577, but not T522, was essential for CK2-dependent HDAC5 regulation. Specifically, overexpression of CSNK2B (CK2β) failed to promote HDAC5 phosphorylation in the HDAC5-T577A mutant, whereas phosphorylation was preserved in the HDAC5-T552A mutant (Figure S9I). Consistent with these findings, the T577A mutation markedly impaired HDAC5 nuclear accumulation following DNA damage and resulted in prolonged Ku70 foci retention at DSB sites (Figure S9J-K).

Collectively, these data indicate that CK2β-mediated phosphorylation of HDAC5 at T577 in response to DNA damage promotes HDAC5 nuclear localization, enables Ku70 deacetylation at K287, and facilitates Ku70 dissociation from DSB sites. Conversely, CK2 inhibition impairs DNA damage-induced HDAC5 translocation, thereby suppressing DNA end resection and HR repair through prolonged Ku70 retention at DSB sites.

### CK2 inhibition sensitizes HDAC5-proficient PDAC to PARP inhibitors

**Figure 6:**
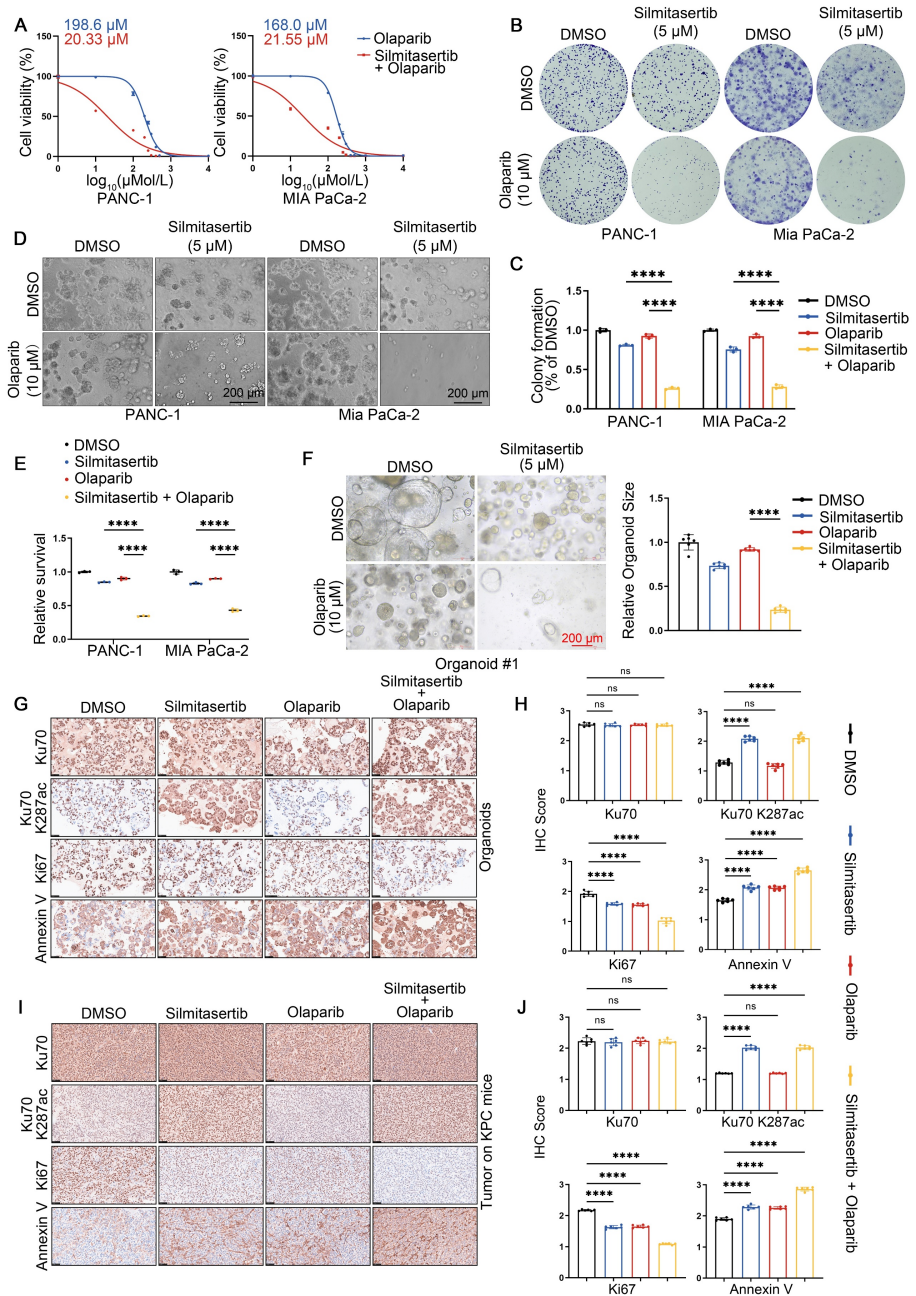
** CK2 inhibition sensitizes HDAC5-proficient PDAC to PARP inhibitors. (A)** Dose-response survival curves of PANC-1 (top) and MIA PaCa-2 (bottom) cell lines treated with escalating olaparib in combination with a fixed 5 μM silmitasertib for 5 days. Data are shown as mean ± SD (n=3 biological replicates). **(B-C)** Colony formation assays were conducted in PANC-1 (left) and Mia PaCa-2 (right) cells treated with DMSO, olaparib (10 μM), silmitasertib (5 μM) or both. Cells were allowed to grow for 14 days before staining. Representative colonies images are shown in **(B)**, with quantification data **(C)**. Data are shown as mean ± SD (n=3 biological replicates). P values were derived from a two-way ANOVA. **(D-E)** 3D cultures were conducted in PANC-1 (left) and Mia PaCa-2 (right) cells treated with DMSO, olaparib (10 μM), silmitasertib (5 μM) or both with samples collected and analyzed following a 7-day treatment period. Scale bar, 200 μm. Representative images are shown in **(D)**, with quantification data **(E)**. Data are shown as mean ± SD (n=3 biological replicates). P values were derived from a two-way ANOVA. **(F)** Representative images (left panel) of human pancreatic cancer organoids treated with DMSO, olaparib (10 μM), silmitasertib (5 μM) or both. Quantification of relative organoid size as represented (right panel). Data are shown as mean ± SD (n=6 biological replicates). P values were derived from a two-way ANOVA. **(G-H)** Representative IHC staining images of Ku70, Ku70 K287ac, Ki67 and Annexin Ⅴ from organoids. Scale bar, 50 μm. Quantification of IHC staining as represented in **(H)**. Data are shown as mean ± SD. P values were derived from a two-way ANOVA. **(I-J)** Representative IHC staining images of Ku70, Ku70 K287ac, Ki67 and Annexin Ⅴ from tumors of KPC mice. Scale bar, 50 μm. Quantification of IHC staining as represented in **(J)**. Data are shown as mean ± SD. P values were derived from a two-way ANOVA. See also Supplementary Figure 10.

Given that pharmacological CK2 inhibition disrupts HDAC5-Ku70 signaling and impairs HR function, we next investigated whether the CK2 inhibitor silmitasertib could sensitize HDAC5-proficient PDAC models to PARP inhibition. Treatment with silmitasertib (5 μM) markedly reduced the IC50 of olaparib from 198.6 μM to 20.33 μM in PANC-1 cells and from 168.0 μM to 21.55 μM in MIA PaCa-2 cells (Figure 6A). Consistent with these findings, colony formation assays and 3D culture experiments demonstrated that silmitasertib (5 μM) significantly enhanced the growth-inhibitory and anti-clonogenic effects of olaparib (10 μM) in both pancreatic cancer cell lines (Figure 6B-E). Similar results were observed in patient-derived pancreatic cancer organoids (PDOs). While olaparib monotherapy (10 μM) had minimal effects on the growth of HDAC5-proficient organoids, co-treatment with silmitasertib (5 μM) resulted in approximately 70% growth inhibition (Figure 6F and Figure S10A). Immunohistochemistry (IHC) analysis of the organoids further showed that silmitasertib co-treatment enhanced olaparib-induced apoptosis and was associated with increased Ku70 K287 acetylation (Figure 6G-H). We further validated these findings in vivo using both a PANC-1 xenografts model (Figure S10B-E) and an autochthonous PDAC model based on LSL-KrasG12D/+; LSL-Trp53R172H/+; Pdx-1-Cre (KPC) mice (Figure 6I, J). Collectively, these data demonstrate that CK2 inhibition with silmitasertib enhances the antitumor efficacy of the PARP inhibitor olaparib in HDAC5-proficient PDAC models.

## Discussion

Upon DNA damage, Ku70 forms a heterodimer with Ku80 and rapidly binds to DSB ends, where it serves as a protective scaffold that limits nuclease accessibility 27, 28, 2, 22, 24. Disruption of the mechanisms governing Ku heterodimer dissociation from DSB sites impairs extensive DNA end resection and compromises efficient HR, particularly during the S phase 23. In this study, we demonstrate that loss of HDAC5 in PDAC, or pharmacological inhibition of CK2, leads to hyperacetylation of Ku70 at lysine 287, resulting in prolonged Ku retention at DSB sites, suppression of HR activity, and enhanced sensitivity to PARP inhibitors.

Ku70 acetylation represents an important post-translational modification that regulates its molecular interactions and biological functions. Acetylation of lysine residues within the C-terminal linker region—such as K533, K539, K542, K544, and K556—has been reported to disrupt the interaction between Ku70 and Bax, thereby facilitating Bax activation and apoptosis initiation 29. Notably, these acetylation events predominantly occur in the cytoplasm and involve monomeric Ku70 as the substrate, potentially following SETD4-mediated methylated and cytoplasmic translocation of Ku70-SAP 30, 31. Similarly, acetylation within the N-terminal α/β domain of Ku70 disrupts the Ku70-FLIP complex, leading to proteasomal degradation of both FLIP and Ku70 and thereby enhancing apoptotic signaling 32, 33. In contrast, acetylation events within the DNA-binding core domain of Ku70 primarily influence its regulatory functions in DSB repair. Acetylation in this region critically regulates Ku70's binding to DSB sites and promotes optimal DSB repair 5, 6, yet the mechanistic details await further clarification. In this study, we identify a previously uncharacterized acetylation site, lysine 287, located within the central DNA-binding core domain of Ku70 and targeted by HDAC5. This residue appears to remain accessible in both monomeric Ku70 and the Ku heterodimer, suggesting a structurally permissive interface for HDAC5-mediated regulation. We further demonstrate that acetylation at K287 promotes prolonged retention of the Ku heterodimer at DSB sites, potentially by altering local charge properties or conformational dynamics within the DNA-binding core domain. An alternative interpretation is that Ku70 deacetylation may be required for efficient NHEJ ligation, such that defective deacetylation leads to impaired NHEJ and a compensatory shift toward HR. However, given the early and transient nuclear accumulation of HDAC5 following DNA damage observed in this study, our data are more consistent with a model in which Ku70 deacetylation facilitates Ku dissociation from DNA ends, thereby permitting DNA end resection. Our data demonstrate that retention of acetylated Ku70 at DSBs suppresses extensive DNA end resection, a prerequisite for the initiation of HR. Consequently, DNA breaks with insufficiently resected ends are channeled into the NHEJ, indicating that the key regulatory function of Ku70 acetylation lies in controlling DNA end resection rather than the ligation process. Our findings establish a nuclear-specific regulatory mechanism whereby Ku70-K287 acetylation fine-tunes DSB repair pathway choice. This mechanism reveals a layered acetylation network wherein distinct lysine residues regulate Ku70 function in a compartment-specific manner, while also highlighting the therapeutic potential of targeting this axis to sensitize tumors to DNA damage-based cancer therapies.

As a member of class IIa histone deacetylase family, HDAC5 is characterized by dynamic nucleocytoplasmic shuttling in response to specific cellular signals 34-36. While the mechanisms by which calcium/calmodulin-dependent protein kinase (CaMK) or protein kinase D (PKD) drive HDAC5 nuclear export have been well characterized, the regulatory pathways controlling its nuclear re-import remain poorly understood 37. In this study, we show that DNA damage-induced phosphorylation of HDAC5 at T577 by CK2 promotes its nuclear localization, thereby enabling HDAC5 to participate in the DNA damage response. This mechanistic insight provides a molecular basis for the observed sensitization to PARP inhibition upon CK2 inhibition.

Consistent with our data, CK2 has been shown to promote NHEJ by activating DNA-PK, though the precise mechanism remains unclear 12. Our study provides a mechanistic explanation for this observation, as stable binding of the Ku heterodimer at DNA double-strand break (DSB) sites constitutes a critical prerequisite for DNA-PK activation 38. Recent studies have further shown that CK2 inhibition can enhance sensitivity to PARP inhibitors by promoting replication fork stalling 13. Together with our findings, these observations suggest that CK2 inhibition may sensitize tumors to PARP inhibition through multiple, non-mutually exclusive mechanisms, including disruption of homologous recombination in HR-proficient contexts.

The copy number of *HDAC5* has been observed to be deleted in a subset of solid tumor patients, including those with prostate cancer and PDAC 15, 17. Our data indicate that HDAC5 deficiency leads to hyperacetylation of Ku70 at K287, resulting in prolonged Ku70 retention at DSB sites and impaired DNA end resection required for efficient HR. These findings suggest that HDAC5 loss may confer an HR-deficient-like state, potentially rendering tumors more sensitive to DNA-damage agents such as platinum compounds or PARP inhibitors 39-41. This warrants further validation through an investigator-initiated clinical study currently under development.

In summary, our study delineates a mechanism by which HDAC5 regulates DSB repair pathway choice through site-specific deacetylation of Ku70 at K287. HDAC5 deficiency results in impaired HR and increased sensitivity to PARP inhibition, while CK2 inhibition phenocopies this effect in HDAC5-proficient settings by disrupting HDAC5 nuclear function. Together, these findings provide a mechanistic framework for understanding PARP inhibitor sensitization beyond canonical HR deficiency.

## Supplementary Material

Supplementary figures and tables.

## Abbreviations

BrdU: 5-bromo-2'-deoxyuridine
CaMK: calcium/calmodulin-dependent protein kinase
CK2: casein kinase 2
Co-IP: Co-Immunoprecipitation
DNA-PK: DNA-dependent protein kinase
DSB: DNA double-strand break
EMSA: electrophoretic mobility shift assays
HDAC5: histone deacetylase 5
HR: homologous recombination
ICC: Immunocytochemistry
IHC: Immunohistochemistry
IR: ionizing radiation
IRIF: ionizing radiation-induced foci
MS: mass spectrometry
NHEJ: non-homologous end joining
PDAC: pancreatic ductal adenocarcinoma
PDOs: patient-derived organoids
PKD: protein kinase D
ssDNA: single-stranded DNA
WB: western blot
WT: wild-type
3D: three-dimensional
4-OHT: 4-Hydroxytamoxifen

## References

[1] Downs JA and Jackson SP A means to a DNA end (2004). the many roles of Ku. Nat Rev Mol Cell Biol.

[2] Shao Z, Davis AJ, Fattah KR, So S, Sun J, Lee KJ (2012). Persistently bound Ku at DNA ends attenuates DNA end resection and homologous recombination. DNA Repair (Amst).

[3] Zahid S, Seif El Dahan M, Iehl F, Fernandez-Varela P, Le Du MH, Ropars V (2021). The Multifaceted Roles of Ku70/80. Int J Mol Sci.

[4] Sharma A, Alswillah T, Kapoor I, Debjani P, Willard B, Summers MK (2020). USP14 is a deubiquitinase for Ku70 and critical determinant of non-homologous end joining repair in autophagy and PTEN-deficient cells. Nucleic Acids Res.

[5] Kim KB, Kim DW, Park JW, Jeon YJ, Kim D, Rhee S (2014). Inhibition of Ku70 acetylation by INHAT subunit SET/TAF-Iβ regulates Ku70-mediated DNA damage response. Cell Mol Life Sci.

[6] Al Emam A, Arbon D, Jeeves M, Kysela B Ku70 N-terminal lysines acetylation/deacetylation is required for radiation-induced DNA-double strand breaks repair Neoplasma. 2018; 65(5): 708-719.

[7] Wang X and Zhao J Targeted Cancer Therapy Based on Acetylation and Deacetylation of Key Proteins Involved in Double-Strand Break Repair Cancer Manag Res. 2022; 14: 259-271.

[8] Subramanian C, Opipari AW Jr, Bian X, Castle VP, Kwok RP Ku70 acetylation mediates neuroblastoma cell death induced by histone deacetylase inhibitors Proc Natl Acad Sci U S A. 2005; 102(13): 4842-4847.

[9] Subramanian C, Hada M, Opipari AW Jr, Castle VP, Kwok RP CREB-binding protein regulates Ku70 acetylation in response to ionization radiation in neuroblastoma Mol Cancer Res. 2013; 11(2): 173-181.

[10] Rabalski AJ, Gyenis L, Litchfield DW Molecular Pathways (2016). Emergence of Protein Kinase CK2 (CSNK2) as a Potential Target to Inhibit Survival and DNA Damage Response and Repair Pathways in Cancer Cells. Clin Cancer Res.

[11] Chen Y, Wang Y, Wang J, Zhou Z, Cao S, Zhang J Strategies of Targeting CK2 in Drug Discovery (2023). Challenges, Opportunities, and Emerging Prospects. J Med Chem.

[12] Olsen BB, Wang SY, Svenstrup TH, Chen BP, Guerra B Protein kinase CK2 localizes to sites of DNA double-strand break regulating the cellular response to DNA damage BMC Mol Biol. 2012; 13: 7.

[13] Ma J, Ren D, Wang Z, Li W, Li L, Liu T (2024). CK2-dependent degradation of CBX3 dictates replication fork stalling and PARP inhibitor sensitivity. Sci Adv.

[14] Mihaylova MM, Vasquez DS, Ravnskjaer K, Denechaud PD, Yu RT, Alvarez JG (2011). Class IIa histone deacetylases are hormone-activated regulators of FOXO and mammalian glucose homeostasis. Cell.

[15] Zhou Y, Jin X, Ma J, Ding D, Huang Z, Sheng H (2021). HDAC5 Loss Impairs RB Repression of Pro-Oncogenic Genes and Confers CDK4/6 Inhibitor Resistance in Cancer. Cancer Res.

[16] Xue Y, Lian W, Zhi J, Yang W, Li Q, Guo X (2019). HDAC5-mediated deacetylation and nuclear localisation of SOX9 is critical for tamoxifen resistance in breast cancer. Br J Cancer.

[17] Pan P, Qin G, Wang B, Yu H, Chen J, Liu J (2022). HDAC5 Loss Enhances Phospholipid-Derived Arachidonic Acid Generation and Confers Sensitivity to cPLA2 Inhibition in Pancreatic Cancer. Cancer Res.

[18] Tyagi W and Das S Temporal regulation of acetylation status determines PARP1 role in DNA damage response and metabolic homeostasis Sci Adv. 2024; 10(42): eado7720.

[19] Gunn A and Stark JM I-SceI-based assays to examine distinct repair outcomes of mammalian chromosomal double strand breaks Methods Mol Biol. 2012; 920: 379-391.

[20] Wang D, Ma J, Botuyan MV, Cui G, Yan Y, Ding D (2021). ATM-phosphorylated SPOP contributes to 53BP1 exclusion from chromatin during DNA replication. Sci Adv.

[21] Wang Y, Czap MS, Kim H, Lu H, Liu J, Chang Y (2024). The Mammalian KU70 C-terminus SAP Domain Is Required to Repair Exogenous DNA Damage. bioRxiv.

[22] Sun J, Lee KJ, Davis AJ, Chen DJ Human Ku70/80 protein blocks exonuclease 1-mediated DNA resection in the presence of human Mre11 or Mre11/Rad50 protein complex J Biol Chem. 2012; 287(7): 4936-4945.

[23] Lee KJ, Saha J, Sun J, Fattah KR, Wang SC, Jakob B (2016). Phosphorylation of Ku dictates DNA double-strand break (DSB) repair pathway choice in S phase. Nucleic Acids Res.

[24] Rinaldi C, Pizzul P, Casari E, Mangiagalli M, Tisi R, Longhese MP The Ku complex promotes DNA end-bridging, this function is antagonized by Tel1/ATM kinase Nucleic Acids Res. 2023; 51(4): 1783-1802.

[25] Chen Y, Wu J, Zhai L, Zhang T, Yin H, Gao H (2024). Metabolic regulation of homologous recombination repair by MRE11 lactylation. Cell.

[26] Lee DY, Lee CI, Lin TE, Lim SH, Zhou J, Tseng YC (2012). Role of histone deacetylases in transcription factor regulation and cell cycle modulation in endothelial cells in response to disturbed flow. Proc Natl Acad Sci U S A.

[27] Lisby M, Barlow JH, Burgess RC, Rothstein R Choreography of the DNA damage response (2004). spatiotemporal relationships among checkpoint and repair proteins. Cell.

[28] Langerak P, Mejia-Ramirez E, Limbo O, Russell P Release of Ku, MRN from DNA ends by Mre11 nuclease activity, Ctp1 is required for homologous recombination repair of double-strand breaks PLoS Genet. 2011; 7(9): e1002271.

[29] Cohen HY, Lavu S, Bitterman KJ, Hekking B, Imahiyerobo TA, Miller C (2004). Acetylation of the C terminus of Ku70 by CBP and PCAF controls Bax-mediated apoptosis. Mol Cell.

[30] Wang Y, Liu B, Lu H, Liu J, Romanienko PJ, Montelione GT (2022). SETD4-mediated KU70 methylation suppresses apoptosis. Cell Rep.

[31] Wang Y, Shen Z Unmasking the mammalian SET domain-containing protein 4 NAR Cancer. 2022; 4(3): zcac021.

[32] Kerr E, Holohan C, Mclaughlin KM, Majkut J, Dolan S, Redmond K (2012). Identification of an acetylation-dependant Ku70/FLIP complex that regulates FLIP expression and HDAC inhibitor-induced apoptosis. Cell Death Differ.

[33] Xiang Z, Hou G, Zheng S, Lu M, Li T, Lin Q (2024). ER-associated degradation ligase HRD1 links ER stress to DNA damage repair by modulating the activity of DNA-PKcs. Proc Natl Acad Sci U S A.

[34] Harrison BC, Huynh K, Lundgaard GL, Helmke SM, Perryman MB, Mckinsey TA Protein kinase C-related kinase targets nuclear localization signals in a subset of class IIa histone deacetylases FEBS Lett. 2010; 584(6): 1103-1110.

[35] Greco TM, Yu F, Guise AJ, Cristea IM Nuclear import of histone deacetylase 5 by requisite nuclear localization signal phosphorylation Mol Cell Proteomics. 2011; 10(2): M110.004317.

[36] Ljubojevic-Holzer S, Herren AW, Djalinac N, Voglhuber J, Morotti S, Holzer M (2020). CaMKIIδC Drives Early Adaptive Ca(2+) Change and Late Eccentric Cardiac Hypertrophy. Circ Res.

[37] Lu J, Mckinsey TA, Zhang CL, Olson EN Regulation of skeletal myogenesis by association of the MEF2 transcription factor with class II histone deacetylases Mol Cell. 2000; 6(2): 233-244.

[38] Zhou C, Li S, Bin K, Qin G, Pan P, Ren D (2022). ITGA2 overexpression inhibits DNA repair and confers sensitivity to radiotherapies in pancreatic cancer. Cancer Lett.

[39] Jamal K, Galbiati A, Armenia J, Illuzzi G, Hall J, Bentouati S (2022). Drug-gene Interaction Screens Coupled to Tumor Data Analyses Identify the Most Clinically Relevant Cancer Vulnerabilities Driving Sensitivity to PARP Inhibition. Cancer Res Commun.

[40] Soung YH and Chung J Combination Treatment Strategies to Overcome PARP Inhibitor Resistance Biomolecules. 2023; 13(10).

[41] Tsujino T, Takai T, Hinohara K, Gui F, Tsutsumi T, Bai X (2023). CRISPR screens reveal genetic determinants of PARP inhibitor sensitivity and resistance in prostate cancer. Nat Commun.

