# Supplementary material for: CK2-mediated HDAC5 shuttling regulates DNA end resection through Ku70 deacetylation
Source: Theranostics. 2026 Jan 8;16(7):3648–64. doi: 10.7150/thno.122935 (PMC12846757; doi:10.7150/thno.122935)
Supplement: Supplementary file 1 — Supplementary figures and tables. [file thnov16p3648s1.pdf]

Supplementary Figures and Legends

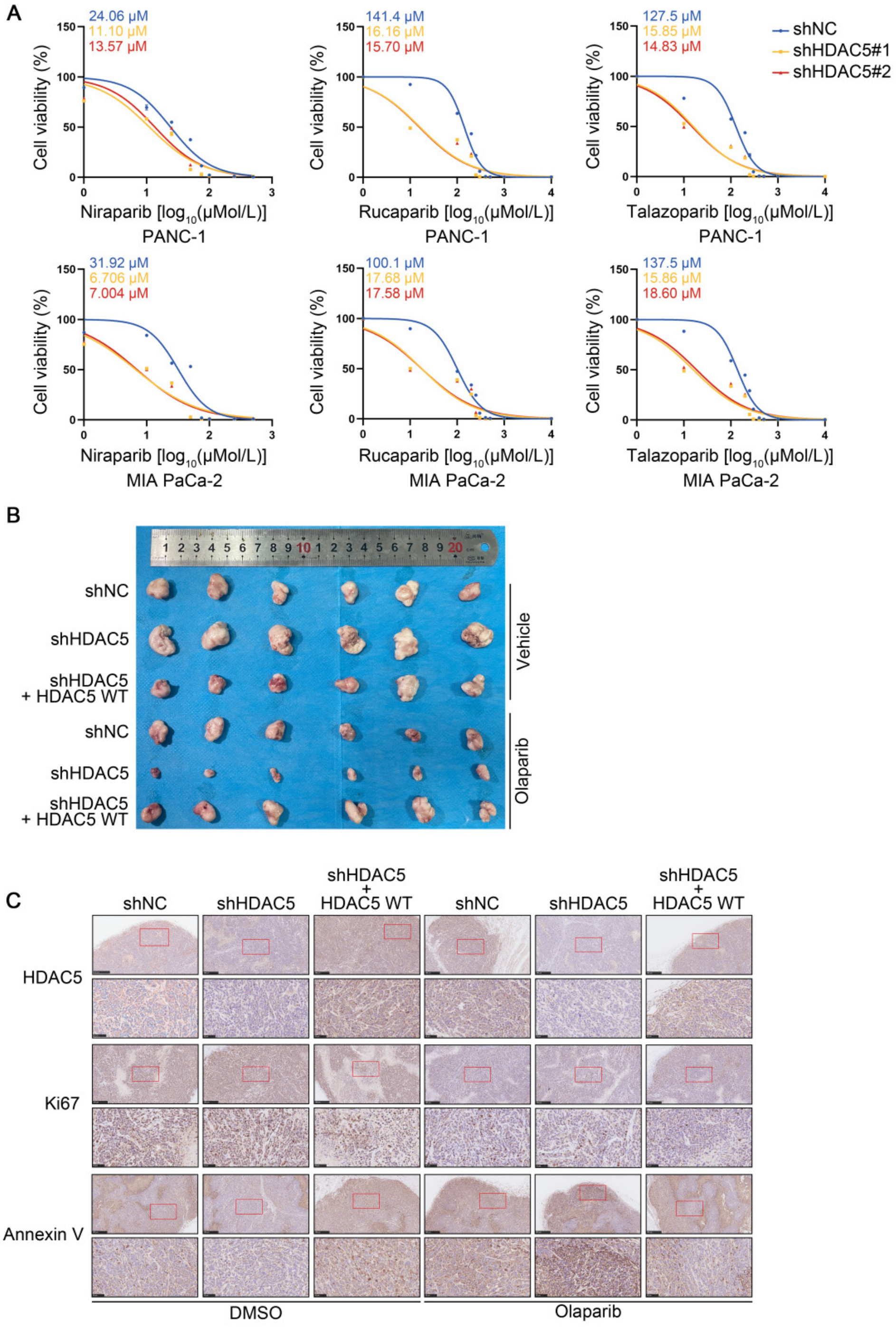

Supplementary Figure 1. HDAC5 loss induces sensitization to PARP inhibitors in homologous recombination proficient PDAC

(A) Dose-response survival curves of shNC, shHDAC5#1, and shHDAC5#2 expressing cells exposed to escalating concentrations of PARP inhibitors in both

PANC-1 (top panel) and MIA PaCa-2 (bottom panel) cell lines. Cell viability was assessed at the end of the 5-day treatment period. Data are shown as mean ± SD (n=3 biological replicates). **(B)** The nude mice were subcutaneously injected with PANC-1 cells expressing shNC, shHDAC5, or shHDAC5+HDAC5 (WT) at the left back. The nude mice were treated with vehicle or olaparib (50 mg/kg, orally) everyday. n=6 mice. Tumor growth was measured every three days for 21 days. Tumors in each group at day 21 were harvested and photographed. Individual tumor are shown. **(C)** Representative IHC staining images of HDAC5, Ki67 and Annexin V from xenograft tumors. Scale bar, 250 and 50 μm.

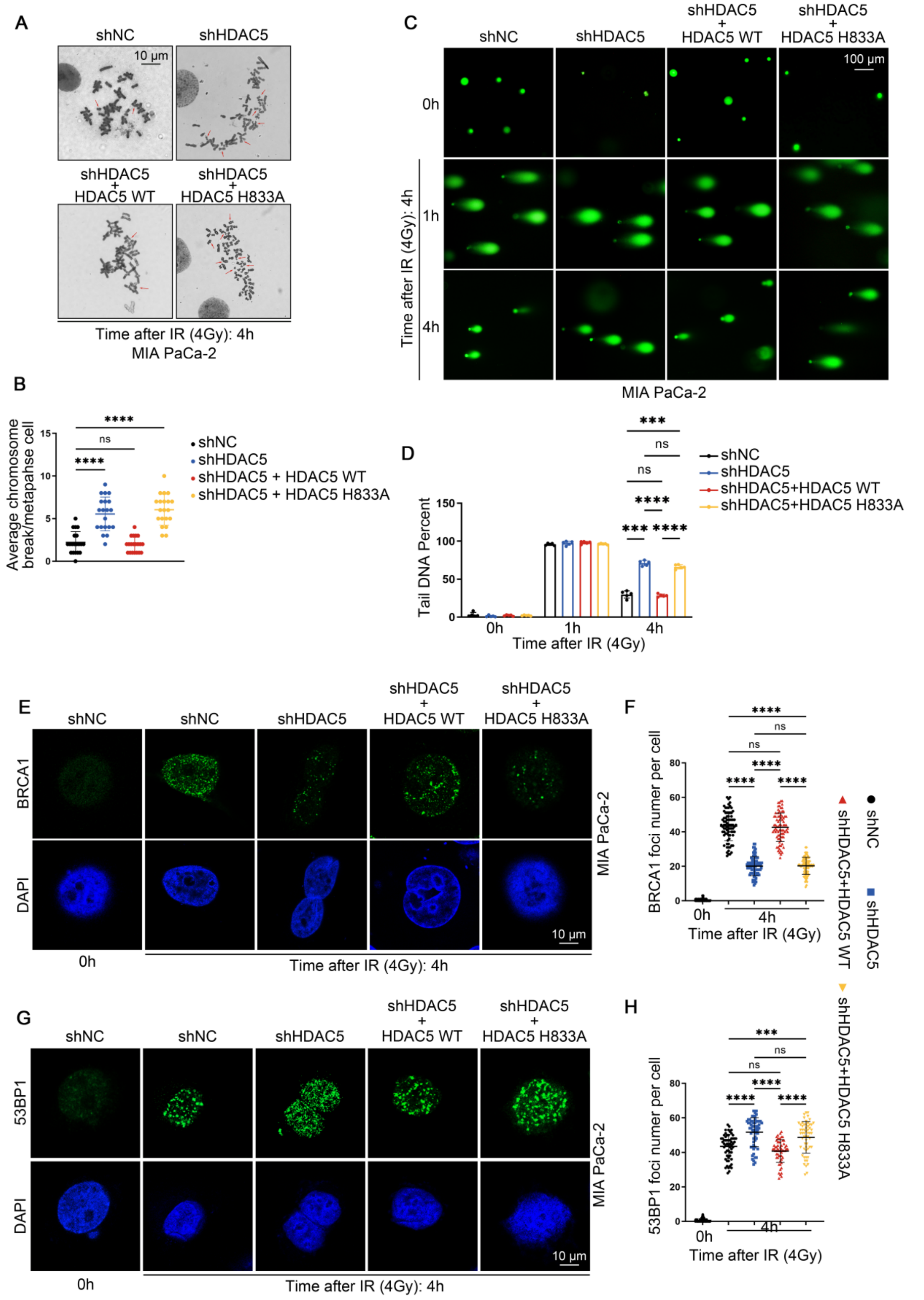

Supplementary Figure 2. HDAC5 maintains HR function in an enzyme-dependent manner

(A-B) Representative karyotypic images showing metaphase chromosome spreads in MIA PaCa-2 cells transfected as indicated, for the purpose of quantifying

chromosomal aberrations. **(A)** Representative aberrations are marked by arrows. Scale bar, 10 μm. **(B)** The average results from three independent experiments with a minimum of 20 cells counted in each experiment. Data were shown as mean ± SD. P values were derived from a one-way ANOVA. **(C-D)** Representative images from comet assays performed under neutral conditions are shown at 1 h and 4 h post-treatment with 4 Gy irradiation, showing delayed repair of DNA in MIA PaCa-2 cells transfected as indicated. **(C)** Representative images show the migration of DNA fragments (comet tail) after irradiation. Scale bar, 100 μm. **(D)** Comet tail DNA precent were quantified from one of three biological replicated (n=5 technical replicates). Data were shown as mean ± SD. P values were derived from a one-way ANOVA. **(E-H)** Representative fluorescence images of BRCA1 **(E)** and 53BP1 **(H)** foci in as indicated MIA PaCa-2 cells. Scale bar, 10 μm. Quantification of the average number of BRCA1 **(G)** and 53BP1 **(I)** foci per focus-positive cell. Data were shown as mean ± SD of more than 50 cells. P values were derived from a two-way ANOVA.

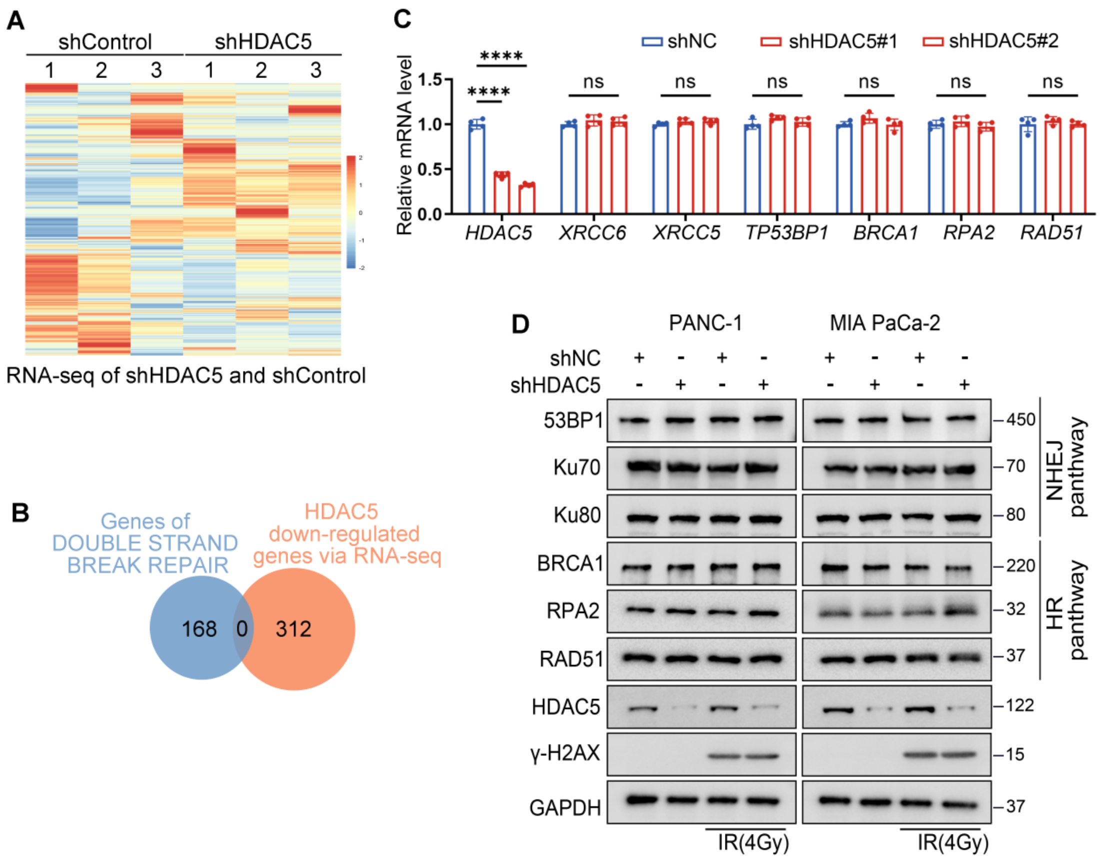

Supplementary Figure 3. HDAC5 maintains HR function in an enzyme-dependent manner

(A) Heatmap showing the result of analysis of RNA-seq between shControl and shHDAC5 in PANC-1 cells. **(B)** Venn diagram showing the overlap of the genes of DSBs repair and the HDAC5 down-regulated genes via RNA-seq. Adj.*p*.val < 0.05. **(C)** RT-qPCR analysis of HDAC5 and DSBs repair-related genes mRNA expression in PANC-1 cells. Data are shown as mean ± SD (n=4 biological replicates). P values were derived from a two-way ANOVA. **(D)** Western blots analysis of HR/NHEJ pathway pivotal protein expression 1 h post-treatment with 4 Gy irradiation in PANC-1 and MIA PaCa-2 cells transfected with shNC or shHDAC5.

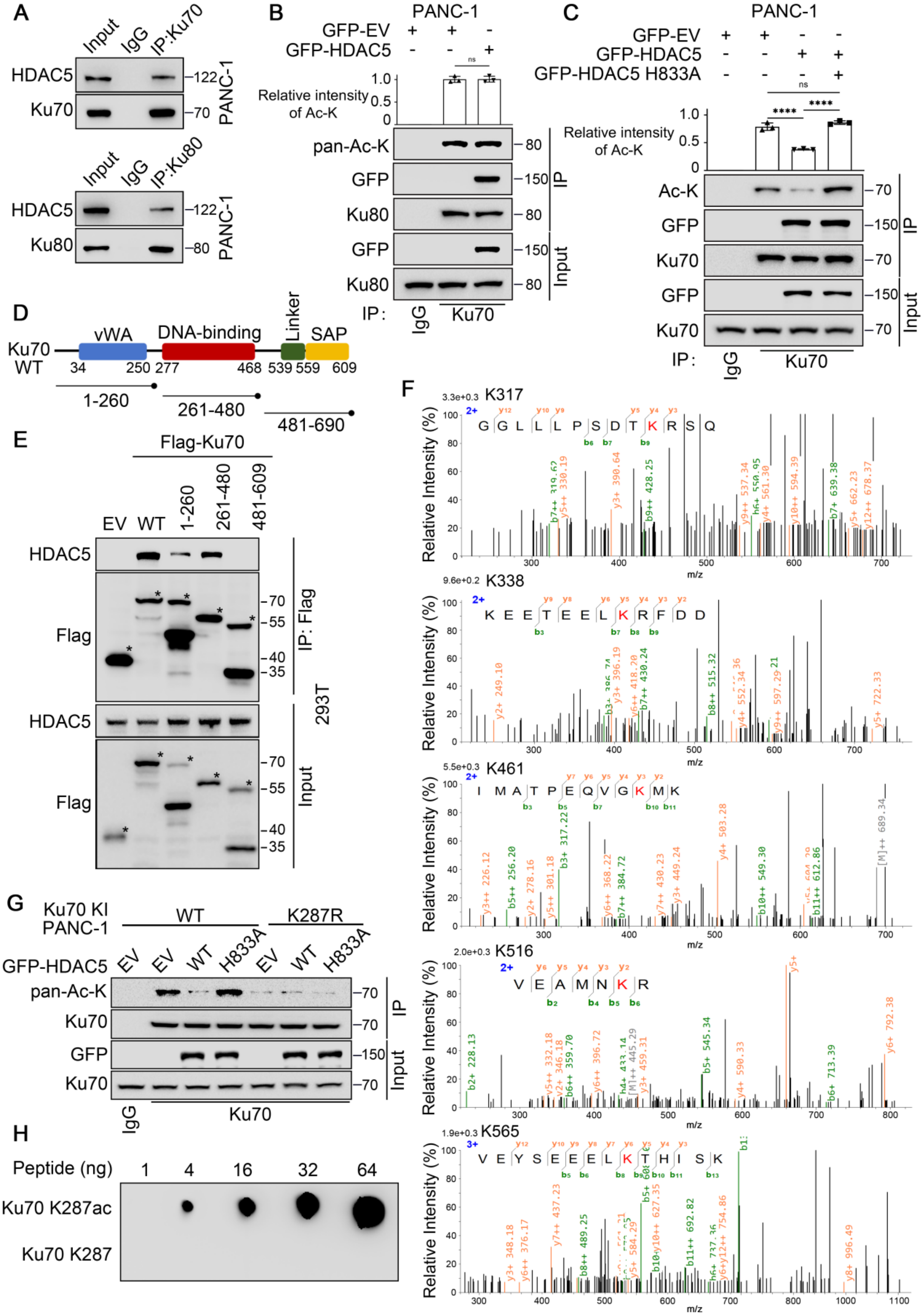

Supplementary Figure 4. HDAC5 regulates DSB repair through site-specific deacetylation of Ku70 at K287

(A) The interaction between endogenous HDAC5 and Ku70 (top panel), and Ku80 (bottom panel) in PANC-1 cells. **(B)** Ku80 acetylation unaffeted by GFP-tagged

HDAC5 overexpression in PANC-1 cells. Relative intensity of acetylation lysine are mean ± SD (n=3 biological replicates). P values were derived from a one-way ANOVA. **(C)** Ku70 acetylation unaffeted upon HDAC5 enzymatically inactivity HDAC5 mutant (H833A) overexpression in PANC-1 cells. Relative intensity of acetylation lysine are mean ± SD (n=3 biological replicates). P values were derived from a one-way ANOVA. **(D)** Schematic model of Ku70 domain structure and constructs. **(E)** The interaction between HDAC5 and the truncations of Ku70 in 293T cells. **(F)** Illustration of Ku70 acetylation at K317, K338, K461, K516 and K565 identified by mass spectrometry. **(G)** Western blot analysis of Ku70 acetylation in Ku70 knock-in PANC-1 cells. **(H)** ddH2O containing different peptides was added onto the nitrocellulose membrane, followed by immunoblotting using anti-Ku70-K287ac antibody.

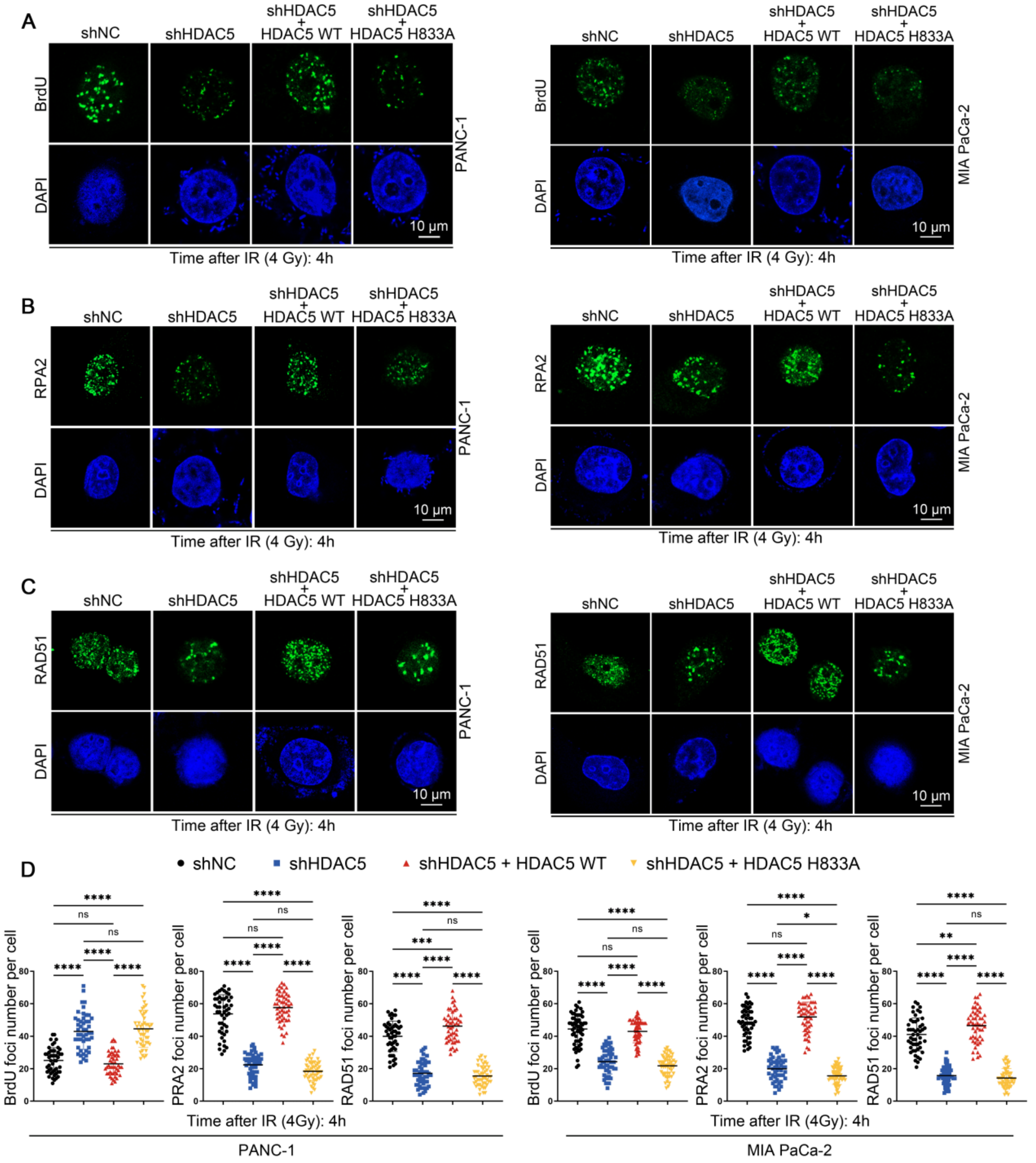

Supplementary Figure 5. HDAC5 deficiency-induced Ku70-K287 acetylation suppresses DNA end resection by prolonging Ku70 retention at DSB sites

(A-C) Representative fluorescence images of BrdU **(A)**, RPA2 **(B)** and RAD51 **(C)** foci in PANC-1 and MIA PaCa-2 cells transfected as indicated. Scale bar, 10 μm. **(D)** Quantification of the average number of BrdU, RPA2 and RAD51 foci per focus-positive cell. Data were shown as mean ± SD of more than 50 cells. P values were derived from a two-way ANOVA.

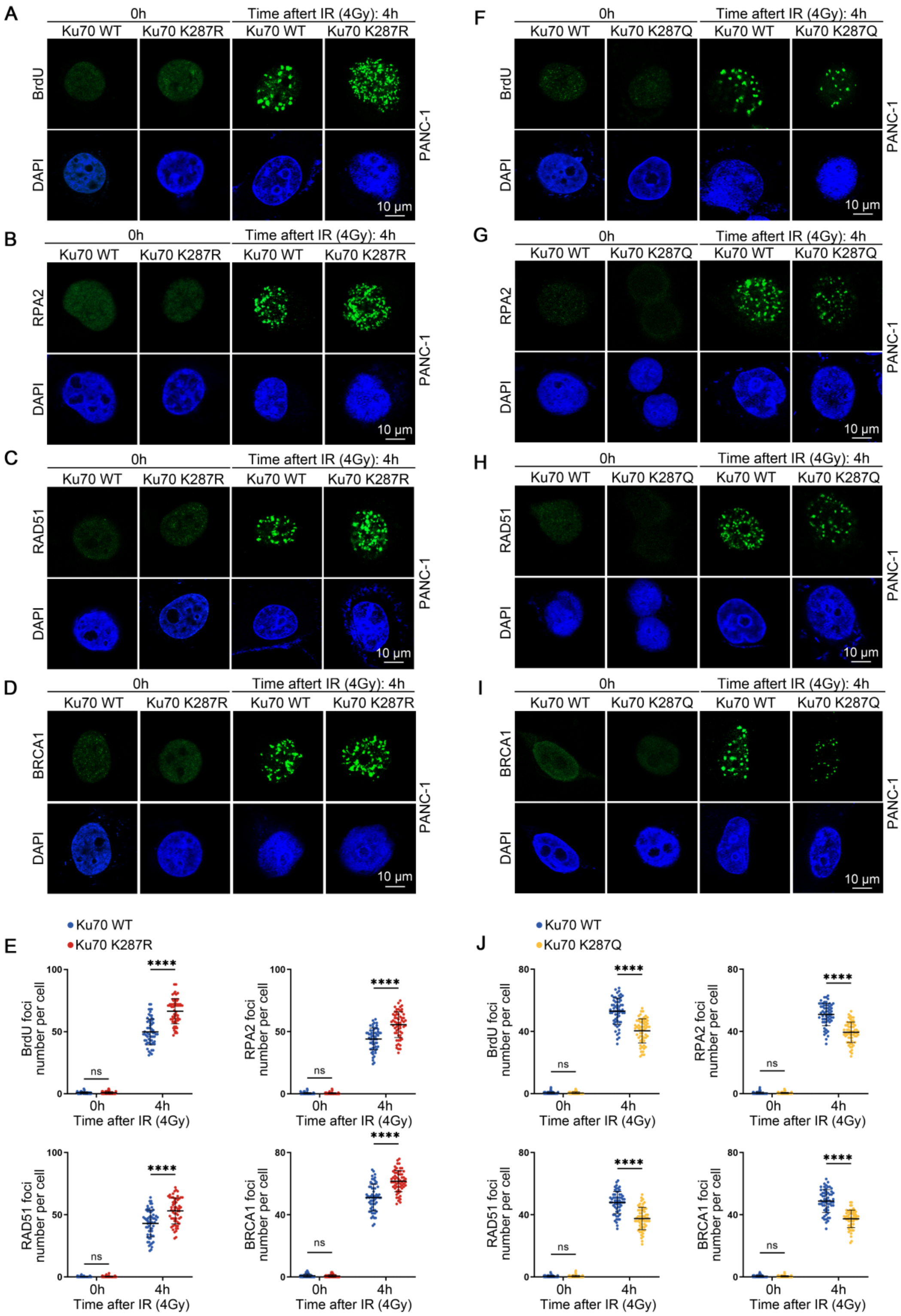

Supplementary Figure 6. HDAC5 deficiency-induced Ku70-K287 acetylation suppresses DNA end resection by prolonging Ku70 retention at DSB sites

(A-D) Representative fluorescence images of BrdU **(A)**, RPA2 **(B)**, RAD51 **(C)** and BRCA1 **(D)** foci in Ku70 Knock-in PANC-1 transfected with WT Ku70 or Ku70

K287R mutant. Scale bar, 10 μm. **(E)** Quantification of the average number of BrdU, RPA2, RAD51 and BRCA1 foci per focus-positive cell. Data were shown as mean ± SD of more than 50 cells. P values were derived from a two-way ANOVA. **(F-I)** Representative fluorescence images of BrdU **(F)**, RPA2 **(G)**, RAD51 **(H)** and BRCA1 **(I)** foci in Ku70 Knock-in PANC-1 transfected with WT Ku70 or Ku70 K287Q mutant. Scale bar, 10 μm. **(J)** Quantification of the average number of BrdU, RPA2, RAD51 and BRCA1 foci per focus-positive cell. Data were shown as mean ± SD of more than 50 cells. P values were derived from a two-way ANOVA.

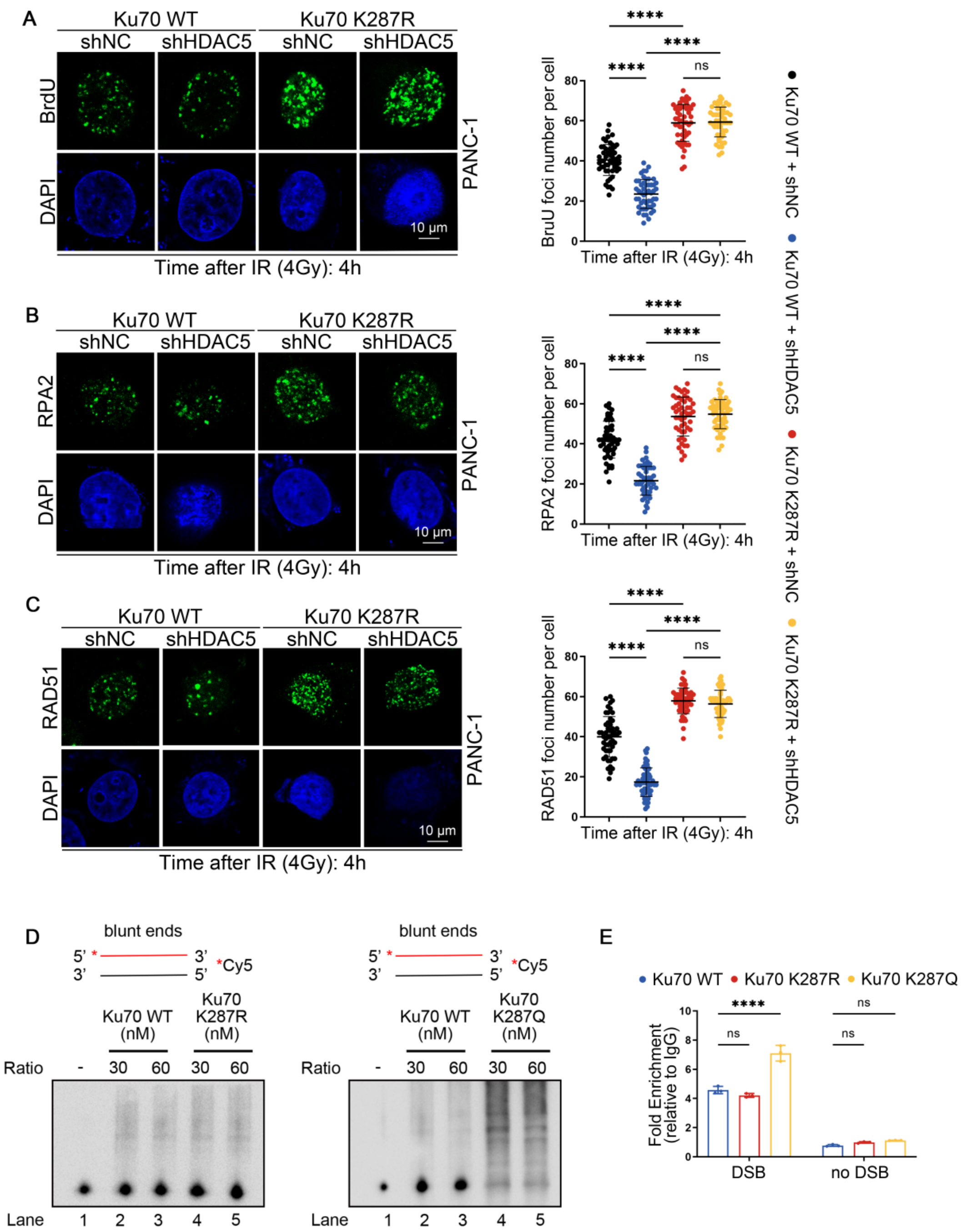

Supplementary Figure 7. HDAC5 deficiency-induced Ku70-K287 acetylation suppresses DNA end resection by prolonging Ku70 retention at DSB sites

(A-C) Representative fluorescence images (left panel) of BrdU **(A)**, RPA2 **(B)** and RAD51 **(C)** foci in PANC-1 transfected as indicated. Scale bar, 10 μm. Quantification

(right panel) of the average number of BrdU, RPA2 and RAD51 foci per focus-positive cell. Data were shown as mean ± SD of more than 50 cells. P values were derived from a two-way ANOVA. **(D)** EMSA was performed using WT Ku70, K287R-mutant and K287Q-mutant proteins, incubated with a Cy5-labeled double-stranded DNA probe. **(E)** ChIP-qPCR analysis of AsiSI-ER U2OS cell lines expressing Ku70 WT, K287-mutant and K287Q-mutant. DSBs were induced by 300 nM 4-OHT for 4 h. Ku70 occupancy was measured at a defined genomic locus proximal to an AsiSI cut site. Data are presented as mean ± SD (n=3 biological replicates). P values were derived from a two-way ANOVA.

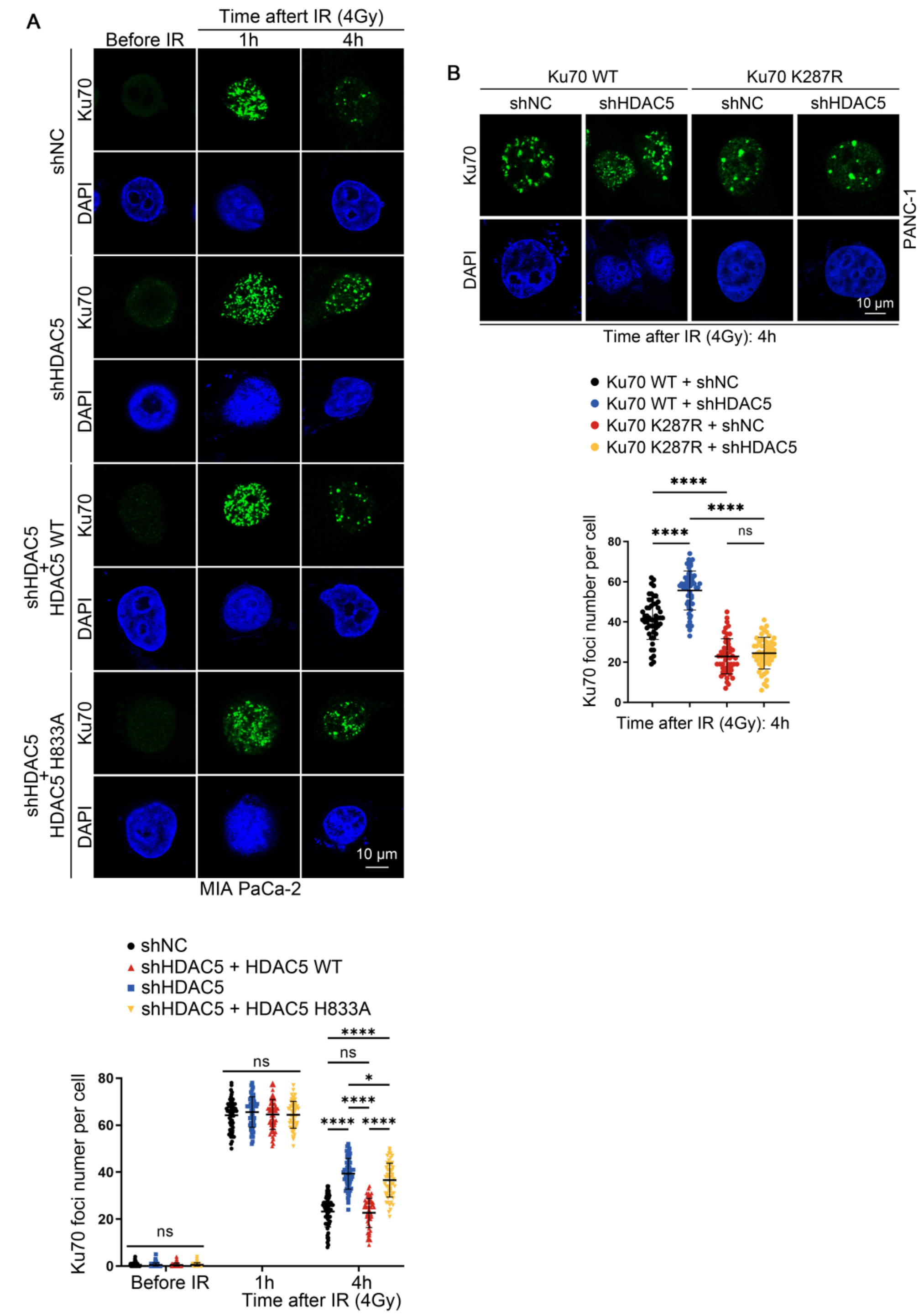

Supplementary Figure 8. HDAC5 deficiency-induced Ku70-K287 acetylation suppresses DNA end resection by prolonging Ku70 retention at DSB sites

(A) Representative fluorescence images (top panel) of Ku70 foci in MIA PaCa-2 cells transfected as indicated. Scale bar, 10 μm. Quantification (bottom panel) of the

average number of Ku70 foci per focus-positive cell. Data were shown as mean ± SD of more than 50 cells. P values were derived from a two-way ANOVA. **(B)** Representative fluorescence images (top panel) of Ku70 foci in PANC-1 transfected as indicated. Scale bar, 10 μm. Quantification (bottom panel) of the average number of Ku70 foci per focus-positive cell. Data were shown as mean ± SD of more than 50 cells. P values were derived from a two-way ANOVA.

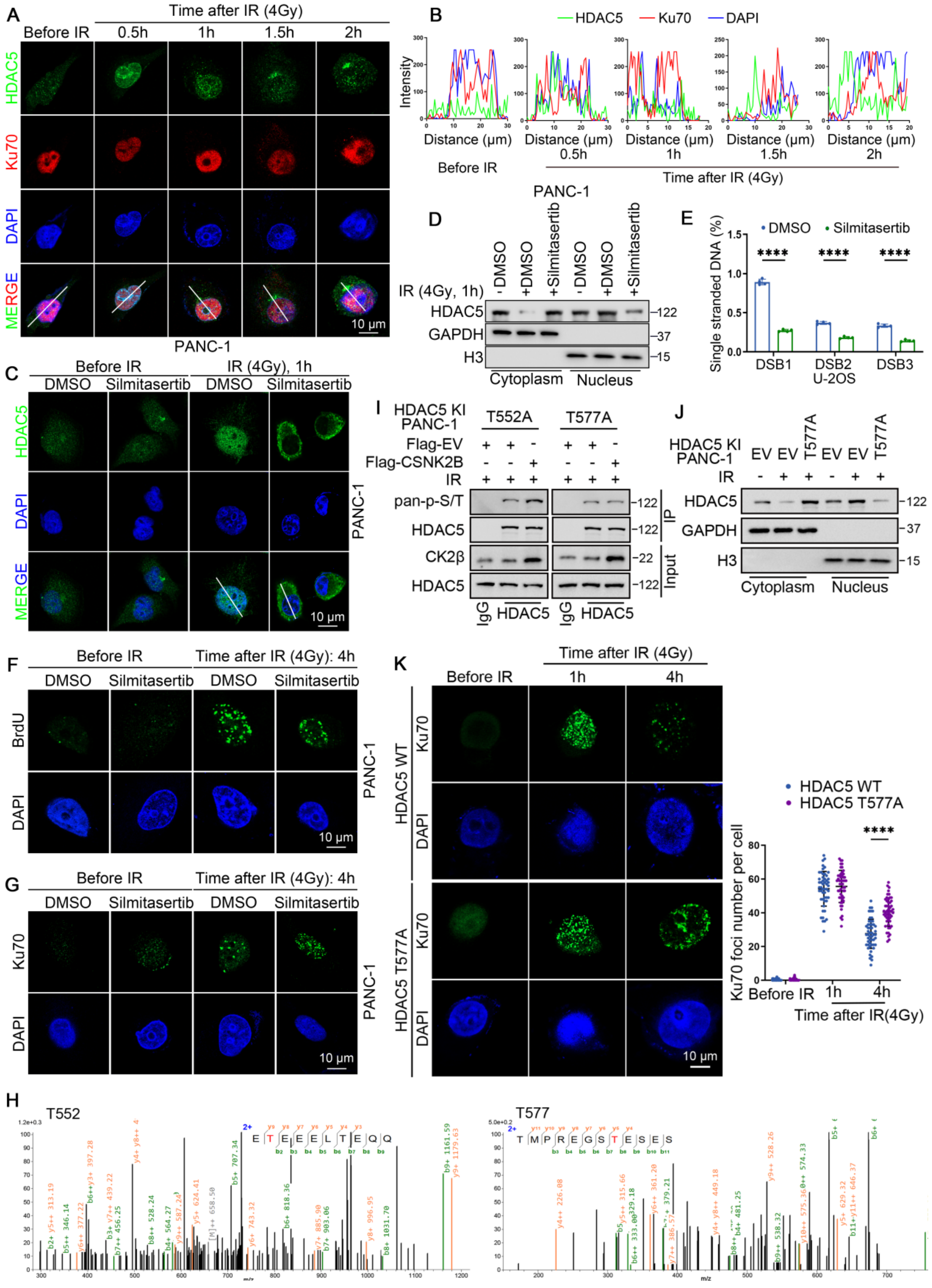

Supplementary Figure 9. CK2 promotes DNA damage-induced nuclear translocation of HDAC5

(A) Representative images of HDAC5 signals (green) and Ku70 (red) in PANC-1 cells treated with different time gradients after 4 Gy irradiation. DAPI, nucleus. Scale

bar, 10μm. **(B)** Plots of pixel intensity along the white line from left to the right of each plot, colors as in merged images. **(C)** Representative immuofluorescence images showing HDAC5 signals (green) in PANC-1 cells. Cells were pre-treated with DMSO or silmitasertib (5 μM) for 48 h, followed by 4 Gy irradiation and fixed 1 h post-irradiation for analysis. DAPI, nucleus. Scale bar, 10μm. **(D)** Western blot analysis of HDAC5 expression in nuclear and cytoplasmic fractions of PANC-1 cells. Cells were pre-treated with DMSO or silmitasertib (5 μM) for 4 h and then harvested 1 h after 4 Gy irradiation. **(E)** RT-qPCR analysis of DNA end resection at designated AsiSI sites in AsiSI-ER U2OS cells. Cells were pre-treated with DMSO or silmitasertib (5 μM) for 4 h. Genomic DNA was harvested 4 h post-4-OHT induction and subjected to the restriction enzyme-based qPCR assay. Data are shown as mean ± SD. P values were derived from a two-way ANOVA. **(F-G)** Representative immunofluorescence images of BrdU **(F)** and Ku70 **(G)** in PANC-1 cells. Cells were pre-treated with DMSO or silmitasertib (5 μM) for 4 h. subjected to 4 Gy irradiation, and fixed 1 h post-irradiation for analysis. DAPI, nucleus. Scale bar, 10 μm. **(H)** Illustration of HDAC5 phosphorylatyion at T552 and T577 identified by mass spectrometry. **(I)** Western blot analysis of HDAC5 phosphorylation. HDAC5 phosphorylation remains unaffected by CSNK2B overexpression in HDAC5 knock-in PANC-1 cells with mutations at T577. **(J)** Western blot analysis of HDAC5 expression in nuclear and cytoplasmic fractions of HDAC5 knock-in PANC-1 cells. **(K)** Representative fluorescence images of Ku70 foci in as indicated PANC-1 cells (left panel). Scale bar, 10 μm. Quantification of the average number of Ku70 fociper focus-positive cell (right panel). Data were shown as mean ± SD of more than 50 cells. P values were derived from a two-way ANOVA.

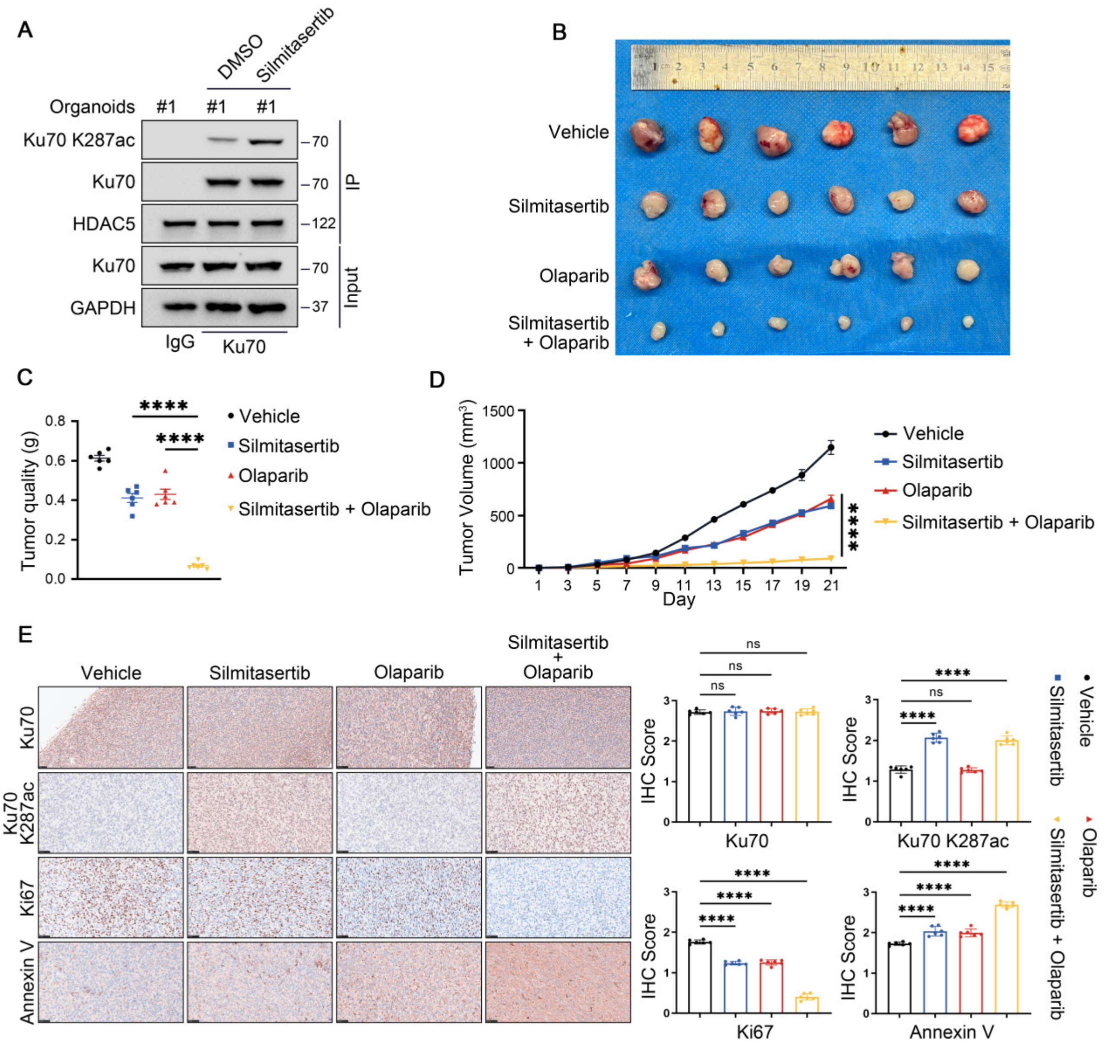

Supplementary Figure 10. CK2 inhibition sensitizes HDAC5-proficient PDAC to PARP inhibitors

(A) Western blot analysis of Ku70 acetylation at the K287 site in human pancreatic cancer organoids. Protein lysates were derived from the organoids shown in Figure 6F after the indicated treatment. **(B-D)** The nude mice were subcutaneously injected with PANC-1 cells at the left back. Mice were randomized into 4 groups of 6 mice: 1) group 1: control group treated with vehicle; 2) group 2: treated with silmitasertib; 3) group 3: treated with olaparib; 4) group 4: treated with silmitasertib and olaparib. All drugs were administered via oral gavages at different concentration: silmitasertib, 75 mg/kg twice daily for 3 weeks; olaparib, 50 mg/kg once daily for 3 weeks. Individual tumors **(B)**, tumor weight **(C)**, and tumor volume **(D)** are shown. Data were shown as mean ± SD. P values were derived from the one-way ANOVA (tumor quality) and the

two-way ANOVA (tumor volume). **(E)** Representative IHC staining images of Ku70, Ku70 K287ac, Ki67 and Annexin V from xenograft tumors. Scale bar, 50 μm. Quantification of IHC staining as represented (right panel). Data are shown as mean ± SD. P values were derived from a two-way ANOVA.

Supplementary Table S1. Sequence of primers and gene specific shRNAs and sgRNAs

Gene	Usage	Forward	Reverse
HDAC5	RT-qPCR	CAGAAGTTGAACGTG GGCAA	GTCCTCCACCAACCT CTTCA
53BP1	RT-qPCR	TGGAAGCTCAGGGA AAGGAG	ACCATCCTCCTCACA CACTG
XRCC6	RT-qPCR	GTTGATGCCTCCAAG GCTATG	CCCCTTAAACTGGTC AAGCTCTA
XRCC5	RT-qPCR	GCACTGACAATCCCC TTTCTG	TCAATGTCCTCCAGC AAATCAAA
BRCA1	RT-qPCR	TGAAGAAAGAGGAA CGGGCT	TGGCTCCCATGCTGT TCTAA
RPA2	RT-qPCR	CGGAGGGAGTAACA CCAACT	TATTGCAGTCCTGAG TCGGG
RAD51	RT-qPCR	GGTCTCTCTGGCAGT GATGT	TCTGTTCTGTAAAGG GCGGT
GAPDH	RT-qPCR	CCATCTTCCAGGAGC GAGAT	GCTGATGATCTTGAG GCTGT
DSB1(364bp)	RT-qPCR	CCAGCAGTAAAGGG GAGACAGA	CTGTTCAATCGTCTG CCCTTC
DSB2(1754bp)	RT-qPCR	GAAGCCATCCTACTC TTCTCACCT	GCTGGAGATGATGAA GCCCA
DSB3(3564bp)	RT-qPCR	GCCCAGCTAAGATCT TCCTTCA	CTCCTTTGCCCTGAG AAGTGA
blunt ends	EMSA	AATTGCAGCTCAGGT CACAGATGCACT	AGTGCATCTGTGACC TGAGCTGCAATT
DSB	ChIP-qPCR	CCAGCAGTAAAGGG GAGACAGA	CTGTTCAATCGTCTG CCCTTC
No DSB	ChIP-qPCR	ATTGGGTATCTGCGT CTAGTGAGG	GACTCAATTACATCC CTGCAGCT
No DSB	RT-qPCR	ATTGGGTATCTGCGT CTAGTGAGG	GACTCAATTACATCC CTGCAGCT
**shRNAs**	**Sequence**		
sh-HDAC5-1	CCGGGACTGTTATTAGCACCTTTAACTCGAGTTAAAGGTGC TAATAACAGTCTTTT		
sh-HDAC5-2	CCGGGCTAGAGAAAGTCATCGAGATCTCGAGATCTCGATG ACTTTCTCTAGCTTTTT		
**SiRNAs**	**Sequence**		
SiCSNK2B	GACAAGCUCUAGACAUGAUTT		
SiTAOK1	GGAGAAACUUAUCAAGAAATT		

SiPIP4K2C	GATGCTGCTGCCAGATGACTT
SiSTK32B	GCTGGATGAACACGGACATGT
**gRNAs**	**Sequence**
gXCRR6 (K287R)	AACCAGTGAAAACCAAGACC
gXCRR6 (K317R)	TAGCGATACCAAGAGGTCTC
gXCRR6 (K338R)	TGAAGAAACACCATTACCTG
gXCRR6 (K461R)	AGAAGCTTCGCTTCACATAC
gXCRR6 (K516R)	CTCCTTGGTGGATGAGTTTA
gXCRR6 (K565R)	CACATCAGCAAGGGTACGCT

Supplementary Table S2. Key Resources

REAGENT or RESOURCE	SOURCE	IDENTIFIER
**Antibodies**		
Mouse polyclonal anti-HDAC5	Abcam	Cat# ab55403; RRID: AB_880353
Rabbit polyclonal anti-Ku70	Proteintech	Cat# 10723-1-AP; RRID: AB_2218756
Rabbit polyclonal anti-Ku80	Proteintech	Cat# 16389-1-AP; RRID: AB_2257509
Rabbit polyclonal anti-BRCA1	Proteintech	Cat# 22362-1-AP; RRID: AB_2879090
Rabbit polyclonal anti-53BP1	Abcam	Cat# ab172580; RRID: AB_2924712
Rabbit polyclonal anti-RAD51	Boster	Cat# A00088; RRID: AB_3080943
Anti-Acetylated-Lysine	Cell Signaling Technology	Cat# 9441; RRID:AB_331805
Rabbit monoclonal anti-Flag-tag	Proteintech	Cat# 66008-4-Ig; RRID:AB_2918475
Rabbit monoclonal anti-GFP-tag	Abclonal	Cat# AE011; RRID:AB_2771922
Rabbit Polyclonal anti-PARP1	Proteintech	Cat# 13371-1-AP; RRID: AB_2160459
Rabbit anti-ADP Ribose	Cell Signaling Technology	Cat# 83732; RRID: AB_2749858
Mouse polyclonal anti-RPA2	Proteintech	Cat# 10412-1-AP; RRID: AB_2269665
Mouse monoclonal anti-BrdU	Abcam	Cat# A300-245A-T; RRID: AB_210547
Rabbit polyclonal anti-GAPDH	Proteintech	Cat# 10494-1-AP; RRID: AB_2263076
Pan Phospho-Serine/Threonine	Abmart	Cat# T91067; RRID: AB_2049269
Rabbit polyclonal anti-Histone H3	Proteintech	Cat# 17168-1-AP; RRID: AB_2716755
Rabbit polyclonal anti-CSNK2B	Proteintech	Cat# 20234-1-AP; RRID: AB_10733246
Rabbit polyclonal anti-Ki67	Proteintech	Cat# 27309-1-AP; RRID: AB_2756525
Rabbit polyclonal anti-AnnexinV	Proteintech	Cat# 11060-1-AP; RRID: AB_2057585
Goat Anti-Rabbit IgG H&L (FITC)	Abcam	Cat# ab6717; RRID: AB_955238

Goat Anti-Mouse IgG H&L (FITC)	Abcam	Cat# ab6785;<br>RRID: AB_955241
Goat Anti-Rabbit IgG H&L (Cy3)	Abcam	Cat# ab6939;<br>RRID: AB_955021
anti-Acetylated Ku70-K287	PTM BioLabs	N/A
**Bacterial and virus strains**		
DH5a Competent *E. coli*	Tsingke	Cat#TSC-C14
BL21 (DE3) Competent *E. coli*	Tsingke	Cat#TSC-E01
**Biological samples**		
Human pancreatic cancer tissue	This paper	N/A
**Chemicals, peptides, and recombinant proteins**		
Polybrene	Beyotime	C0351
Puromycin Dihydrochloride	Beyotime	ST551
Colcemid	MedChem Express	HY-N0282R
Potassium chloride (KCl)	Solarbio	P9921
Bromodeoxyuridine	Selleck	S7918
4-Hydroxytamoxifen (4-OHT)	Selleck	S7827
BamHI	Thermo Fisher Scientific	ER0051
Olaparib	Selleck	S1060
Silmitasertib	Selleck	S2248
Recombinant Ku70 protein	Abcam	ab132938
Recombinant Ku80 protein	Abcam	ab132946
**Critical commercial assays**		
FDA-approved Anticancer Drug Library	Selleck	L8000
GST-tag Protein Purification Kit	Beyotime	P2260S
GST Protein Interaction Pull-Down Kit	Thermo Fisher Scientific	2156
Diff-Quick Stain Kit	Solarbio	G1541
Cell Counting Kit-8	Beyotime	C0037
**Experimental models: Cell lines**		
HEK293T	Procell	Cat# CL-0005<br>RRID: CVCL_0063
PANC-1	Procell	Cat#CL-0184<br>RRID: CVCL_0480
MIA PaCa-2	Procell	Cat#CL-0627<br>RRID: CVCL_0428
U2OS	Procell	Cat#CL-0236<br>RRID: CVCL_0042
**Experimental models: Organisms/strains**		
Mouse: BALB/C Nude Mice	Beijing Vitalriver Laboratory Animal	Cat#401<br>RRID: MGI: 2161072

	Technology	
Mouse: KPC transgenic mice (*SL-KrasG12D/+; LSL-Trp53R172H/+; Pdx-1-Cre*; 8-week-old)	Cyagen	N/A
**Recombinant DNA**		
GFP-HDAC5	Genechem	N/A
Flag-Ku70		
Software and algorithms		
Graphpad Prism 9.5	Graphpad software	http://www.graphpad.com/
ImageJ	ImageJ: Image Processing and Analysis in Java	https://imagej.nih.gov/ij/
